# Association of Lipoprotein(a) Levels With Incidence of Major Adverse Limb Events

**DOI:** 10.1001/jamanetworkopen.2022.45720

**Published:** 2022-12-08

**Authors:** Alexis F. Guédon, Jean-Baptiste De Freminville, Tristan Mirault, Nassim Mohamedi, Bastien Rance, Natalie Fournier, Jean-Louis Paul, Emmanuel Messas, Guillaume Goudot

**Affiliations:** 1 Vascular Medicine Department, Georges Pompidou European Hospital, Assistance Publique–Hôpitaux de Paris (APHP), Université Paris Cité, Paris, France; 2 Paris Cardiovascular Research Center (PARCC), Institut National de la Santé et de la Recherche Médicale (INSERM) U970, Université Paris Cité, Paris, France; 3 Department of Medical Informatics, Georges Pompidou European Hospital, APHP, Université Paris Cité, Paris, France; 4 Biology Department, Biochemistry Laboratory, Georges Pompidou European Hospital, APHP, Université Paris Cité, Paris, France; 5 Lip(Sys)2-EA7357, Athérosclérose et Macrophages: Impact Des Phospholipides e Des Fonctions Mitochondriales Sur l'efflux du Cholestérol Cellulaire, Université Paris-Saclay, UFR de Pharmacie, Chatenay-Malabry, France

## Abstract

**Importance:**

High lipoprotein(a) (Lp[a]) levels are involved in the development of cardiovascular events, particularly in myocardial infarction, stroke, and peripheral artery disease. Studies assessing the Lp(a) levels associated with adverse lower-limb events are lacking.

**Objective:**

To assess the association between Lp(a) levels and incidence of major adverse limb events in unselected hospitalized patients.

**Design, Setting, and Participants:**

This large retrospective monocentric cohort study was conducted from January 1, 2000, to December 31, 2020. Data were derived from the clinical information system of the Hôpital Européen Georges-Pompidou, a Paris-based university hospital. Patients who underwent at least 1 Lp(a) measurement at the center during the study period were included. Patients who had no follow-up data or who had the first Lp(a) measurement after the study outcome had occurred were excluded. Data analyses were performed from May 2021 to January 2022.

**Main Outcomes and Measures:**

The primary outcome was the first inpatient major adverse limb event, defined as a major amputation, peripheral endovascular revascularization, or peripheral surgical revascularization, during follow-up. Secondary outcomes included individual components of the primary outcome. Lipoprotein(a) levels were categorized as follows: normal (<50 mg/dL), high (50 to <134 mg/dL), and very high (≥134 mg/dL); to convert Lp(a) values to milligrams per liter, multiply by 0.1.

**Results:**

A total of 16 513 patients (median [IQR] age, 58.2 [49.0-66.7] years; 9774 men [59.2%]) were included in the cohort. The median (IQR) Lp(a) level was 24 (10.0-60.0) mg/dL. The 1-year incidence of major adverse limb event was 2.44% in the overall population and 4.54% among patients with very high Lp(a) levels. High (adjusted accelerated failure time [AFT] exponential estimate: 0.43; 95% CI, 0.24-0.78; Benjamini-Hochberg–corrected *P* = .01) and very high (adjusted AFT exponential estimate: 0.17; 95% CI, 0.07-0.40; Benjamini-Hochberg–corrected *P* < .001) Lp(a) levels were independently associated with an increased risk of major adverse limb event.

**Conclusions and Relevance:**

Results of this study showed that higher Lp(a) levels were independently associated with an increased risk of a major adverse limb event in hospitalized patients. The Lp(a) measurement needs to be taken into account to improve lower-limb vascular risk assessment.

## Key Points

**Question:**

What is the association between lipoprotein(a) (Lp[a]) levels and major adverse limb events, defined as major amputation, peripheral artery endovascular revascularization, or peripheral artery surgical revascularization?

**Finding:**

In this cohort study of 16 513 patients, patients with high Lp(a) levels had a major adverse limb events–free survival that was 57% shorter than in patients with normal Lp(a) levels. Patients with very high Lp(a) levels had a major adverse limb event–free survival that was 83% shorter than that of patients with normal levels.

**Meaning:**

Findings of this study suggest that higher Lp(a) levels are independently associated with an increased risk of major adverse limb events and that Lp(a) measurement might help improve lower-limb vascular risk assessment.

## Teaser

This cohort study investigates whether lipoprotein(a) values are associated with lower-limb amputation or revascularization.

## Introduction

Peripheral artery disease (PAD) is an atherosclerotic disease of the lower-limb arteries affecting more than 200 million people worldwide.^1,2^ Moreover, PAD remains underdiagnosed and untreated due to the asymptomatic onset of the disease.^3^ Patients with PAD can develop limb symptoms, ranging from claudication to critical limb-threatening ischemia, leading to a major adverse limb event, such as peripheral artery revascularization and lower-limb amputation.^1,4,5,6^ In addition to the usual cardiovascular risk factors, such as diabetes, arterial hypertension, and smoking, one of the most important indicators of major adverse limb event is a previous peripheral revascularization itself, highlighting the importance of preventing the occurrence of major adverse limb events.^7,8,9^

Lipoprotein(a) (Lp[a]) is a low-density lipoprotein (LDL)-like particle synthesized in the liver and is composed of an apolipoprotein B100 covalently bound to the apolipoprotein(a), a glycoprotein not found in native LDL.^10^ High Lp(a) levels are well described in the literature as being involved in the development of cardiovascular events,^11,12,13,14,15,16,17,18,19,20,21^ such as myocardial infarction (MI) and stroke. Underlying pathophysiological mechanisms have not yet been fully elucidated and have a converging association with the development of atherosclerosis and thrombosis.^22,23,24,25,26,27^ More than 90% of Lp(a) plasmatic levels are genetically determined, with low influence from the environment or nutrition,^10^ and statins have a neutral or modest Lp(a)-increasing outcome.^28^ New treatments are currently being developed to specifically reduce this target.^29^ Thus far, Lp(a) levels are assessed to better stratify patients at intermediate cardiovascular risk.^30^ Furthermore, elevated Lp(a) levels are associated with an increased risk of PAD, but studies investigating the role of Lp(a) in lower-limb amputation or revascularization in a large population are lacking.^31,32,33,34^ To better understand the potential value of Lp(a)-lowering therapy in PAD, we assessed the association between Lp(a) levels and the incidence of major adverse limb events in unselected hospitalized patients.

## Methods

This study received approval from the CERAPHP.5 Institutional Review Board. Because the study was retrospective and observational, consent from patients was not required by French law. The study was performed in accordance with the Declaration of Helsinki.^35^ We followed the Strengthening the Reporting of Observational Studies in Epidemiology (STROBE) reporting guideline.^36^

### Data Collection and Study Population

Data were derived from the clinical information system of the Hôpital Européen Georges-Pompidou (HEGP), a Paris-based university hospital.^37^ Since its opening in 2000, HEGP has used a certified clinical information system,^38^ combined with an i2b2 clinical data warehouse, to facilitate the reuse of health care data.^39,40^ All clinical data were coded using the *International Statistical Classification of Diseases and Related Health Problems, Tenth Revision* (*ICD-10*), and all procedures performed during the hospital stay were coded using CCAM (Classification Commune des Actes Médicaux), the French common classification of medical procedures.^41,42^

**Figure 1.**
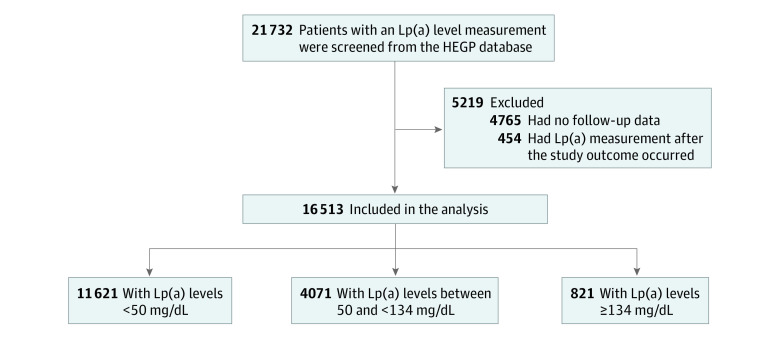
Flowchart of Study Population HEGP indicates Hôpital Européen Georges-Pompidou; Lp(a), lipoprotein(a).

All patients (n = 21 732) who underwent at least 1 Lp(a) measurement at HEGP between January 1, 2000, and December 31, 2020, were screened. The routine lipid panel testing at HEGP always includes Lp(a) measurement. Patients who had no follow-up data (n = 4765) or who underwent the first Lp(a) measurement after the primary outcome had occurred (n = 454) were excluded (Figure 1). We considered the date of the first Lp(a) measurement to be the inclusion date and the date of the final hospital contact or date of the outcome occurrence to be the last follow-up date.

### Lipoprotein(a) Measurement

All venous blood samples were collected during the hospital stay. Quantitation of Lp(a) levels was performed by nephelometry technique using rabbit polyclonal anti–Lp(a) antibody. All measurements were carried out using an immunochemistry system (IMMAGE 800; Beckman Coulter). The Lp(a) measurements were reported in milligrams per deciliter; to convert to milligrams per liter, multiply by 0.1. Measurement of this assay ranges from 2 to 640 mg/dL.

### Outcomes and Covariates

Age, sex, hospitalization data, and biological variables were extracted from electronic medical records using the HEGP clinical information system. Race and ethnicity data were not collected because ethnic statistics are forbidden in France by the Data Protection Act of January 6, 1978. Comorbidities were identified from the *ICD-10* codes related to the hospitalization at the inclusion date. The primary outcome was the first inpatient major adverse limb event, defined as a major amputation, peripheral endovascular revascularization, or peripheral surgical revascularization, during follow-up. We defined major amputation as a lower-limb amputation event above the forefoot. Peripheral endovascular and peripheral surgical procedures included endovascular and surgical revascularization, respectively, of lower-limb arteries or aorta. Secondary outcomes were the individual components of the primary outcome. Corresponding CCAM procedure codes and *ICD-10* comorbidity codes are presented in eMethods 1 and 2 in Supplement 1. Lipid-lowering treatment data were not available for patients who were admitted for 1-day hospitalization. Distribution of patients by hospitalization unit and Lp(a) levels at inclusion are shown in eFigure 5 in Supplement 1.

### Statistical Analysis

Continuous variables were described as medians (IQRs), and binary variables were expressed as numbers (proportions). Qualitative variables were compared using Fisher exact tests, and binary variables were compared using Kruskal-Wallis tests. We computed Lp(a) level categories less than 50 mg/dL as the upper limit for a normal level, according to the 2018 American College of Cardiology and American Heart Association Guideline on the Management of Blood Cholesterol^43^ and the European Atherosclerosis Society consensus statement about Lp(a).^44^ The 95th percentile of Lp(a) level (ie, ≥134 mg/dL in the present cohort) was used to define a very high level, as proposed in the literature.^45,46,47,48^ Lp(a) levels between 50 and less than 134 mg/dL were used to define a high level. Lp(a), LDL-cholesterol, and serum creatinine levels were log-transformed in the multivariate regression analyses due to the positively skewed distribution.

We used Kaplan-Meier curves to generate outcome-free survival curves and compute 1-year, 5-year, and 10-year cumulative incidence rates of outcomes. Given that the Schoenfeld residuals analyses showed a violation of the proportional hazards assumption required for Cox proportional hazards regression models,^49^ we used accelerated failure time (AFT) regression models to assess the association between Lp(a) levels and outcomes. Accelerated failure time models assume that an explanatory variable acts multiplicatively on the speed at which an event occurs for a patient.^50,51^ Among log-normal, log-logistic, Weibull, exponential, gaussian, and logistic distributions, we chose the log-normal distribution, which provided the best model fit according to the Akaike information criterion. The exponentiated AFT regression coefficient represents acceleration factors. An AFT exponential estimate of 1.0 shows no association between the explanatory variable and the outcome. Values lower than 1.0 show an earlier occurrence of the event, and values higher than 1.0 show delayed occurrence of the event. We fit multivariate AFT models to analyze the association between primary or secondary outcomes and log-transformed Lp(a) levels or Lp(a) threshold. Sex, age, diabetes, arterial hypertension, LDL-cholesterol level, smoking status, serum creatinine level, and dialysis were considered as potential confounders, according to the literature, and were included in the regression models.^3,52,53^ All AFT models with included variables and coefficient results are shown in eTables 5-8 in Supplement 1.

We performed sensitivity analyses with the primary outcome models, adding lipid-lowering drug status for patients with available data (eTable 1 in Supplement 1). The time effect variable was defined as the binary variable inclusion time before or after the median inclusion time of follow-up for this cohort, which was set to September 29, 2011 (eTable 2 in Supplement 1). Multiple imputation with chained equations to handle missing data was performed at baseline using the MICE package^54^ in R, version 3.5.0 (R Foundation for Statistical Computing). Estimates were pooled across 10 imputed data sets.

To reduce α risk inflation, we used the Benjamini-Hochberg false discovery rate approach^55^ to obtain corrected *P* values for all tests involving the primary outcome, including sensitivity analyses (eTable 9 in Supplement 1). Two-sided testing was used, with Benjamini-Hochberg–corrected *P* < .05 considered to be statistically significant. All analyses were performed using R, version 3.6.0, for Mac (R Foundation for Statistical Computing). Data analyses were performed from May 2021 to January 2022.

## Results

### Patient Population and Baseline Characteristics

A total of 16 513 patients were included. These patients had a median (IQR) age of 58.2 (49.0-66.7) years and included 6739 women (40.8%) and 9774 men (59.2%). Among these patients, 10 502 (63.6%) had dyslipidemia; 9062 (54.9%) had arterial hypertension; 2146 (13.0%) had diabetes; and approximately one-third were either former smokers (2770 [16.8%]) or current smokers (2394 [14.5%]) (Table 1).

**Table 1.** Characteristics of Study Participants and by Lp(a) Level Categories^a^

	No. (%)				*P* value
	Overall (n = 16 513)	Normal level (n = 11 621)	High level (n = 4071)	Very high level (n = 821)	
Age, median (IQR), y	58.2 (49.0-66.7)	58.0 (48.6-66.7)	58.3 (49.6-66.7)	60.1 (51.9-67.8)	<.001
Female sex	6739 (40.8)	4528 (39.0)	1790 (44.0)	421 (51.3)	<.001
Male sex	9774 (59.2)	7093 (61.0)	2281 (56.0)	400 (48.7)	<.001
PAD	1139 (6.9)	726 (6.2)	313 (7.7)	100 (12.2)	<.001
History of ischemic heart disease	2387 (14.5)	1548 (13.3)	681 (16.7)	158 (19.2)	<.001
History of stroke	204 (1.2)	122 (1.0)	65 (1.6)	17 (2.1)	.002
Arterial hypertension	9062 (54.9)	6310 (54.3)	2255 (55.4)	497 (60.5)	.002
Cigarette use					
Never	11 349 (68.7)	7824 (67.3)	2899 (71.2)	626 (76.2)	<.001
Former	2770 (16.8)	2017 (17.4)	647 (15.9)	106 (12.9)	
Current	2394 (14.5)	1780 (15.3)	525 (12.9)	89 (10.8)	
Dyslipidemia	10 502 (63.6)	7126 (61.3)	2810 (69.0)	566 (68.9)	<.001
Diabetes	2146 (13.0)	1480 (12.7)	521 (12.8)	145 (17.7)	<.001
Dialysis	75 (0.5)	52 (0.4)	17 (0.4)	6 (0.7)	.47
Lp(a)					
Median (IQR), mg/dL	24 (10-60)	15 (7-27)	78 (62-10)	168 (150-202)	
By level category	16 513 (100.0)	11 621 (70.4)	4071 (24.7)	821 (5.0)	
Cholesterol level, median (IQR), mg/dL					
LDL	118.33 (92.42-146.56)	117.56 (91.65-144.62)	120.26 (94.35-150.43)	120.65 (93.19-156.23)	<.001
HDL	48.72 (39.83-59.94)	48.34 (39.06-59.55)	49.50 (40.99-60.71)	52.20 (42.54-64.19)	<.001
Total	194.51 (165.12-227.38)	193.35 (163.96-225.06)	197.60 (167.44-231.63)	201.08 (170.92-242.07)	<.001
Triglyceride level, median (IQR), mg/dL	106.19 (76.11-153.10)	107.08 (76.11-155.75)	104.42 (75.22-146.90)	107.08 (78.76-156.64)	.002
Serum creatinine level, median (IQR), mg/dL	0.90 (0.76-1.10)	0.90 (0.76-1.09)	0.92 (0.76-1.12)	0.96 (0.77-1.26)	<.001
Measured GFR, median (IQR), mL/min/1.73 m^2^	80 (66-93)	80 (67-93)	78 (64-92)	72 (53-89)	<.001
Proteinuria, median (IQR), g/L	0.12 (0.08-0.18)	0.12 (0.08-0.18)	0.12 (0.08-0.18)	0.12 (0.07-0.23)	.24
Conjugated bilirubin level, median (IQR), mg/dL	0.23 (0.18-0.29)	0.23 (0.18-0.29)	0.23 (0.18-0.29)	0.18 (0.12-0.23)	.71
Total bilirubin level, median (IQR), mg/dL	0.70 (0.53-0.88)	0.70 (0.53-0.94)	0.70 (0.53-0.88)	0.64 (0.18-0.29)	<.001
γ-glutamyltransferase level, median (IQR), U/L	22 (14-37)	22 (13-38)	22 (14-37)	23 (14-40)	.51
Alkaline phosphatase level, median (IQR), U/L	60 (50-74)	60 (49-73)	62 (51-76)	67 (54-83)	<.001
Alanine aminotransferase level, median (IQR), U/L	21 (15-30)	21 (15-31)	21 (15-29)	18 (13-26)	<.001
Aspartate aminotransferase level, median (IQR), U/L	21 (17-26)	21 (17-26)	21 (17-26)	21 (17-26)	.64
Serum albumin, median (IQR), g/dL	3.8 (3.4-4.1)	3.8 (3.5-4.1)	3.7 (3.2-4.0)	3.7 (3.0-3.9)	<.001
Lipid-lowering drugs, No./total No. (%)	1520/2852 (53.3)	944/1901 (49.7)	435/749 (58.1)	141/202 (69.8)	<.001
Follow-up duration, median (IQR), y	3.74 (1.07-7.30)	3.80 (1.11-7.35)	3.64 (1.01-7.27)	3.29 (1.08-6.74)	.02

Abbreviations: GFR, glomerular filtration rate; HDL, high-density lipoprotein; LDL, low-density lipoprotein; Lp(a), lipoprotein(a); PAD, peripheral artery disease.

SI conversion factors: To convert alkaline phosphatase to microkatal per liter, multiply by 0.0167; ALT and AST to microkatal per liter, multiply by 0.0167; bilirubin to micromoles per liter, multiply by 17.104; γ-glutamyltransferase to microkatal per liter, multiply by 0.0167; LDL, HDL, and total cholesterol to millimoles per liter, multiply by 0.0259; Lp(a) to milligrams per liter, multiply by 0.1; serum albumin to grams per liter, multiply by 10; serum creatinine to micromoles per liter, multiply by 88.4; triglycerides to millimoles per liter, multiply by 0.0113.

^a^ Continuous variables were described as medians (IQRs), and binary variables were expressed as numbers (proportions). Qualitative variables were compared using Fisher tests, and binary variables were compared using Kruskal-Wallis tests. Lipoprotein(a) level categories were as follows: normal (<50 mg/dL), high (50 to <134 mg/dL), and very high (≥134 mg/dL).

Lipoprotein(a) levels ranged from 2 to 540 mg/dL, and the median (IQR) Lp(a) level was 24.0 (10.0-60.0) mg/dL. A total of 11 621 patients (70.4%) were within the normal level (<50 mg/dL), 4071 patients (24.7%) were within the high level (50 to <134 mg/dL), and 821 patients (5.0%) were within the very high level (≥134 mg/dL). Distribution is shown in eFigure 1 in Supplement 1. Compared with patients with high or normal Lp(a) level categories, patients with a very high level were less frequently men (56.0% or 61.0% vs 48.7%; *P* < .001). Patients with a very high level had more comorbidities, notably PAD (12.2% vs 7.7% or 6.2%; *P* < .001) and ischemic heart disease (19.2% vs 16.7% or 13.3%; *P* < .001) compared with those with high or normal Lp(a) levels. Concomitantly, patients with a very high level had higher total cholesterol, HDL-cholesterol, or LDL-cholesterol levels than those within high or normal Lp(a) levels (Table 1). Further details regarding subgroup characteristics are available in eTables 3 and 4 in Supplement 1.

### Association of High and Very High Lp(a) Levels With Major Adverse Limb Event

**Figure 2.**
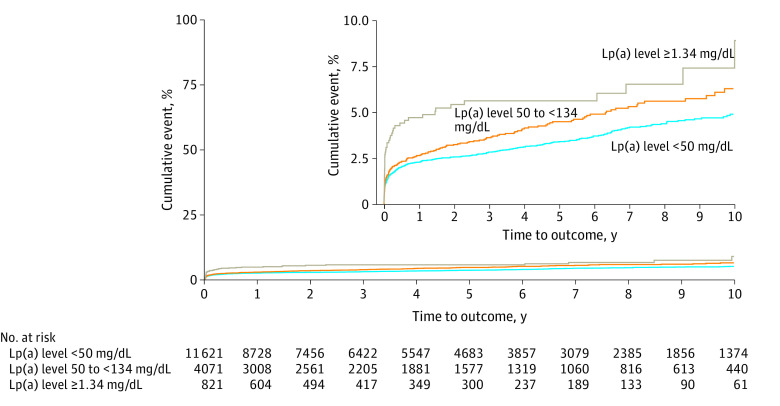
Kaplan-Meier Survival Curves of the Cumulative Incidence of Major Adverse Limb Event (MALE) by Lipoprotein(a) (Lp[a]) Level Categories The inset shows the detail on an enlarged y-axis.

Cumulative incidence of major adverse limb event and its individual components are presented in Table 2. A total of 572 major adverse limb events occurred during a median (IQR) follow-up period of 3.74 (1.07-7.30) years. The cumulative incidence rates were 2.44% (95% CI, 2.20%-2.68%) for 1 year, 3.70% (95% CI, 3.37%-4.03%) for 5 years, and 5.33% (95% CI, 4.80%-5.85%) for 10 years. The 1-year incidence rate of major adverse limb event was 4.54% (95% CI, 3.08%-5.98%) among patients with very high Lp(a) levels, and the 1-year major amputation rate overall was 0.40% (95% CI, 0.30%-0.50%). Figure 2 shows Kaplan-Meier survival curves according to the Lp(a) level categories. Patients with very high Lp(a) level had a 5-year cumulative incidence of 5.44% (95% CI, 3.79%-7.07%) vs 4.43% (95% CI, 3.68%-5.16%) for those with high level or 3.33% (95% CI, 2.96%-3.70%) for those with normal level.

**Table 2.** Cumulative Incidence of Primary and Secondary Outcomes by Lp(a) Level Categories^a^

Lp(a) level category	Patients, No.	1-y Event rate		5-y Event rate		10-y Event rate	
		Event, No.	Rate (95% CI), %	Event, No.	Rate (95% CI), %	Event, No.	Rate (95% CI), %
**Major adverse limb event**							
Overall	16 513	383	2.44 (2.20-2.68)	504	3.70 (3.37-4.03)	572	5.33 (4.80-5.85)
Normal	11 621	247	2.24 (1.96-2.51)	320	3.33 (2.96-3.70)	366	4.83 (4.24-5.41)
High	4071	100	2.60 (2.09-3.10)	143	4.43 (3.68-5.16)	161	6.21 (5.06-7.34)
Very high	821	36	4.54 (3.08-5.98)	41	5.44 (3.79-7.07)	45	8.67 (4.72-12.45)
**Major amputation**							
Overall	16 513	61	0.40 (0.30-0.50)	80	0.59 (0.46-0.73)	90	0.84 (0.63-1.04)
Normal	11 621	38	0.35 (0.24-0.47)	49	0.5 (0.36-0.65)	56	0.76 (0.51-1.00)
High	4071	19	0.50 (0.28-0.73)	24	0.76 (0.44-1.07)	27	1.00 (0.57-1.42)
Very high	821	4	0.56 (0.01-1.11)	7	1.10 (0.28-1.93)	7	1.10 (0.28-1.93)
**Endovascular revascularization**							
Overall	16 513	288	1.83 (1.62-2.04)	377	2.78 (2.49-3.07)	429	4.08 (3.6-4.54)
Normal	11 621	180	1.63 (1.39-1.87)	233	2.44 (2.12-2.76)	267	3.56 (3.05-4.07)
High	4071	76	1.97 (1.53-2.41)	108	3.36 (2.71-4.01)	124	5.16 (4.02-6.30)
Very high	821	32	4.03 (2.65-5.38)	36	4.82 (3.24-6.38)	38	6.19 (3.66-8.65)
**Surgical revascularization**							
Overall	16 513	114	0.74 (0.60-0.88)	162	1.23 (1.04-1.43)	198	2.11 (1.75-2.47)
Normal	11 621	77	0.71 (0.55-0.87)	109	1.17 (0.94-1.39)	133	1.98 (1.57-2.38)
High	4071	29	0.76 (0.48-1.03)	44	1.41 (0.98-1.84)	52	2.18 (1.46-2.89)
Very high	821	8	1.09 (0.33-1.85)	9	1.28 (0.44-2.12)	13	4.15 (0.68-7.51)

Abbreviation: Lp(a), lipoprotein(a).

^a^ Lipoprotein(a) level categories were as follows: normal (<50 mg/dL), high (50 to <134 mg/dL), and very high (≥134 mg/dL).

Unadjusted and adjusted AFT analysis showed that Lp(a) was associated with increased incidence of a major adverse limb event (adjusted AFT exponential estimate: 0.57; 95% CI, 0.46-0.71; Benjamini-Hochberg–corrected *P* < .001) (Table 3). Sensitivity analyses confirmed this result, by adjusting on lipid-lowering drug (adjusted AFT exponential estimate: 0.69; 95% CI, 0.53-0.89; Benjamini-Hochberg–corrected *P* < .001); history of ischemic heart disease (adjusted AFT exponential estimate: 0.59; 95% CI, 0.47-0.73; Benjamini-Hochberg–corrected *P* < .001); history of stroke (adjusted AFT exponential estimate: 0.57; 95% CI, 0.46-0.71; Benjamini-Hochberg–corrected *P* < .001); or PAD, a major factor associated with major adverse limb event (adjusted AFT exponential estimate: 0.82; 95% CI, 0.69-0.97; Benjamini-Hochberg–corrected *P* = .03).

**Table 3.** AFT Survival Model With Lognormal Distribution for the Full Cohort^a^

Outcome	Variable	Nonadjusted AFT model exponential estimate (95% CI)	*P* value		Adjusted AFT model exponential estimate (95% CI)^b^	*P* value	
			Uncorrected	Benjamini-Hochberg–corrected		Uncorrected	Benjamini-Hochberg–corrected
Major adverse limb event	Log[Lp(a)]	0.61 (0.48-0.76)	<.001	<.001	0.57 (0.46-0.71)	<.001	<.001
	High Lp(a)^c^	0.50 (0.27-0.93)	.03	.04	0.43 (0.24-0.78)	.005	.008
	Very High Lp(a)^c^	0.18 (0.07-0.44)	<.001	<.001	0.17 (0.07-0.40)	<.001	<.001
Major amputation	Log[Lp(a)]	0.56 (0.35-0.91)			0.62 (0.40-0.98)		
	High Lp(a)^c^	0.42 (0.12-1.41)			0.41 (0.13-1.32)		
	Very High Lp(a)^c^	0.22 (0.03-1.96)			0.41 (0.05-3.40)		
Endovascular revascularization	Log[Lp(a)]	0.49 (0.37-0.65)			0.49 (0.37-0.63)		
	High Lp(a)^c^	0.41 (0.20-0.84)			0.36 (0.18-0.70)		
	Very High Lp(a)^c^	0.07 (0.02-0.24)			0.09 (0.03-0.29)		
Surgical revascularization	Log[Lp(a)]	0.80 (0.60-1.07)			0.72 (0.54-0.95)		
	High Lp(a)^c^	0.74 (0.34-1.61)			0.61 (0.28-1.30)		
	Very high Lp(a)^c^	0.45 (0.11-1.89)			0.35 (0.09-1.45)		
Major adverse limb event							
Sensitivity analyses with lipid-lowering drug when prescription was available (n = 2852) as adjustment variable	Log[Lp(a)]				0.69 (0.53-0.89)	.004	.007
Sensitivity analyses with PAD added as adjustment variable	Log[Lp(a)]				0.82 (0.69-0.97)	.02	.03
Sensitivity analyses with history of ischemic heart disease added as adjustment variable	Log[Lp(a)]				0.59 (0.47-0.73)	<.001	<.001
Sensitivity analyses with history of stroke added as adjustment variable	Log[Lp(a)]				0.57 (0.46-0.71)	<.001	<.001

Abbreviations: AFT, accelerated failure time; Lp(a), lipoprotein(a); PAD, peripheral artery disease.

^a^ Some cells were purposely left blank to indicate that tests were not performed to avoid α risk inflation, as stated in Methods.

^b^ AFT regressions were adjusted on sex, age, diabetes, arterial hypertension, low-density lipoprotein cholesterol, smoking status, serum creatinine levels, and dialysis. AFT regression subanalyses with lipid-lowering drugs available included the same covariables, with the lipid-lowering drugs status added.

^c^ Compared with normal Lp(a) level category as the reference.

Regarding Lp(a) level categories, patients within the very high level had a major adverse limb event–free survival time that was 83% shorter than that of patients within the normal level over a median (IQR) follow-up period of 3.29 (1.08-6.74) years (adjusted AFT exponential estimate: 0.17; 95% CI, 0.07-0.40; Benjamini-Hochberg–corrected *P* < .001). Accordingly, patients within the high Lp(a) level had a major adverse limb event–free survival time that was 57% shorter than that of patients within the normal level over a median (IQR) follow-up period of 3.64 (1.01-7.27) years (adjusted AFT exponential estimate: 0.43; 95% CI, 0.24-0.78; Benjamini-Hochberg–corrected *P* = .01).

Regarding secondary outcomes, 90 major amputations, 429 endovascular revascularizations, and 198 surgical revascularizations occurred during follow-up. Adjusted AFT analysis suggested that Lp(a) was associated with major amputation (adjusted AFT exponential estimate: 0.62; 95% CI, 0.40-0.98), endovascular revascularization (adjusted AFT exponential estimate: 0.49; 95% CI, 0.37-0.63), and surgical revascularization (adjusted AFT exponential estimate: 0.72; 95% CI, 0.54-0.95). On the other hand, none of the Lp(a) level categories was associated with major amputation or surgical revascularization (Table 3). Nevertheless, patients with very high (adjusted AFT exponential estimate: 0.09; 95% CI, 0.03-0.29) or high Lp(a) levels (adjusted AFT exponential estimate: 0.36; 95% CI, 0.18-0.70) tended to have more endovascular revascularization than patients with a normal Lp(a) level. Kaplan-meier survival curves of secondary outcomes incidence are presented in eFigures 2-4 in Supplement 1.

## Discussion

In this large, retrospective cohort study using administrative data from a single center in France, we found that the Lp(a) level was independently associated with an increased incidence of major adverse limb event. High Lp(a) level (values between 50 and <134 mg/dL) was associated with an increased risk of major adverse limb event compared with a normal Lp(a) level (<50 mg/dL), corresponding to a 0.43 times shorter time to event (or 57% shorter major adverse limb event–free survival over the median follow-up period of 3.64 years). Very high Lp(a) level (≥134 mg/dL) was associated with an increased risk of major adverse limb event compared with a normal Lp(a) level, corresponding to a 0.17 times shorter time to event (or 83% shorter major adverse limb event–free survival over the median follow-up period of 3.29 years).

Lipoprotein(a) level increase is known to be associated with an increased occurrence of cardiovascular events.^11,12,13,14,15,16,17,18,19,20,21^ Many studies have investigated the association between MI and Lp(a) and reported an increased risk in patients with elevated Lp(a) values.^11,12,13,56,57^ Previous studies have suggested that Lp(a) could also be associated with stroke.^14,15,16^ Regarding PAD, the InCHIANTI (Invecchiare in Chianti) study involving 1002 Italian patients showed a cross-sectional association between Lp(a) levels and PAD prevalence.^58^ Similarly, in the Edinburgh Artery Study, elevated Lp(a) values corresponding to an increased intertertile range were significantly associated with PAD, with an adjusted hazard ratio (HR) of 1.22 (95% CI, 1.04-1.44).^59^ Moreover, the prospective European Prospective Investigation into Cancer–Norfolk study including 18 720 patients found an adjusted HR for PAD of 1.37 (95% CI, 1.25-1.50) for a 2.7-fold increase in Lp(a) levels.^57^ Recently, an Australian study including 1472 patients with PAD, which was defined by intermittent claudication, abdominal aortic aneurysm, or critical limb ischemia, found that Lp(a) levels higher than 30 mg/dL were associated with more requirements for any PAD intervention (lower-limb peripheral revascularization, abdominal aortic aneurysm repair, other aneurysm repair, or carotid artery revascularization), with an HR of 1.33 (95% CI, 1.06-1.66).^33^

Though data are scarce on the elevated Lp(a) values associated with the incidence of major adverse limb event,^31,32^ we believe we provided such data in this study, which assessed the association between Lp(a) and major adverse limb event in, to our knowledge, the largest cohort of unselected inpatient population. An analysis of the Spanish FRENA registry reported that among the 1503 stable outpatients with coronary, cerebrovascular, or peripheral artery disease, patients with Lp(a) levels between 30 and 50 mg/dL had significantly more limb amputations than those with Lp(a) levels less than 30 mg/dL (HR, 3.18; 95% CI, 1.36-7.44) and even more amputations were required for those with Lp(a) levels higher than 50 mg/dL (HR, 22.7; 95% CI, 9.38-54.9).^34^ A prespecified analysis of the ODYSSEY OUTCOMES (Evaluation of Cardiovascular Outcomes After an Acute Coronary Syndrome During Treatment With Alirocumab) randomized clinical trial conducted in 18 924 patients with recent acute coronary syndrome found in the placebo group a significant link between Lp(a) levels and risk of PAD events, which were defined as any critical limb ischemia, limb revascularization, or amputation for ischemia.^60^ Two other studies found an association between elevated Lp(a) levels and major adverse limb event incidence among patients with documented PAD who underwent iliofemoral endarterectomy or endovascular therapy.^61,62^

These previous findings are consistent with the results of the current study, wherein Lp(a) values between 50 mg/dL and less than 134 mg/dL or 134 mg/dL or greater were associated with a major adverse limb event–free survival time that was 57% or 83% shorter, respectively, than the time for a Lp(a) level less than 50 mg/dL. In this cohort, the 1-year major adverse limb event rate was 2.44%, and the 1-year major amputation rate was 0.40%. In the COMPASS (Cardiovascular Outcomes for People Using Anticoagulation Strategies) randomized, double-blind placebo-controlled trial assessing the efficacy of low-dose rivaroxaban and aspirin combination or rivaroxaban alone compared with aspirin alone, the incidence of major adverse limb event was 1.2% per year, and the incidence of major amputation was 0.4% per year in the aspirin with placebo group.^8,63^ In the EUCLID (Examining Use of Ticagrelor in PAD) trial comparing clopidogrel with ticagrelor in patients with symptomatic PAD, the incidence of major adverse limb event was 0.77 per 100 person-year, and the incidence of major amputation was 0.41 per 100 person-year.^64,65^ In the COMPASS trial, however, the major adverse limb event outcome was defined as acute limb ischemia, chronic limb ischemia, and major amputation, whereas in the EUCLID trial, the definition included only acute limb ischemia and major amputation. We could not differentiate between acute and chronic revascularizations due to the coding modalities of the HEGP medicoadministrative database, and thus we could not distinguish acute limb ischemia from chronic limb-threatening ischemia interventions. Despite this situation, the major adverse limb event rate seemed higher than rates observed in other studies probably due to a recruitment bias from the HEGP. However, the major amputation rate was consistent with rates observed in previous trials.

The Lp(a) level thresholds we selected may be debated. We chose less than 50 mg/dL as the upper limit of normal level to be in line with the 2018 American College of Cardiology and American Heart Association Guideline on the Management of Blood Cholesterol and the European Atherosclerosis Society consensus statement about Lp(a),^44^ in which a level greater than 50 mg/dL was considered to be a factor associated with enhanced risk of atherosclerotic cardiovascular disease. For the choice of the threshold to define extreme Lp(a) levels, the 2019 European Society of Cardiology/European Atherosclerosis Society Guidelines for the Management of Dyslipidaemias recommended considering 1 lifetime Lp(a) measurement to detect patients with extreme Lp(a) levels (>180 mg/dL).^66^ This guideline was based on a Mendelian randomization study suggesting that extreme Lp(a) levels increased atherosclerotic cardiovascular disease risk similarly to the levels in people with heterozygous familial hypercholesterolemia.^67^ Because a recent study did not have enough power to assess the association between extreme Lp(a) levels and the need for a peripheral artery revascularization,^33^ we took advantage of the large size of the present cohort to establish the 95th percentile Lp(a) level (≥134 mg/dL) as the very high level category, which was proposed in several studies.^45,46,47,48^ The very high level category was associated with major adverse limb event risk in this cohort. Moreover, the association between Lp(a) and major adverse limb event incidence remained significant after adjustment for history of ischemic heart disease or history of stroke in the sensitivity analyses, and with PAD being the main factor associated with major adverse limb event. Beyond indicating the Lp(a) level associated with MI and coronary heart disease,^47^ these results reinforce the potential association of Lp(a) with improved lower-limb vascular risk.

The pathophysiological mechanisms underlying the atherogenicity of Lp(a) remain poorly elucidated. However, 3 central components can be described: prothrombotic (plasminogen-like), proatherogenic (LDL-like), and proinflammatory^10,22^ properties. First, due to the homologic structure of apolipoprotein(a) with plasminogen, high Lp(a) levels interfere with the intrinsic fibrinolysis, promoting thrombosis and increasing cardiovascular events.^23,24^ Second, as for LDL cholesterol, it has been shown in atheromatous coronary and carotid lesions that there is an accumulation of Lp(a) through its LDL particle components, suggesting a deposit on atherosclerotic lesions and an increased risk of plaque rupture.^25,26^ In addition, Lp(a) can have a proinflammatory role in the endothelium via the oxidized phospholipids bound to the apolipoprotein(a).^27^ A recent secondary analysis of the ACCELERATE (Assessment of Clinical Effects of Cholesteryl Ester Transfer Protein Inhibition With Evacetrapib in Patients at a High Risk for Vascular Outcomes) trial investigating the effects of evacetrapib on patients with high cardiovascular risk reported that elevated Lp(a) levels were associated with cardiovascular death, MI, or stroke only when high-sensitivity C-reactive protein levels were greater than 2 mg/mL.^68^ As much as an elevated Lp(a) level seems associated with the development of coronary atherosclerosis through Lp(a) genetic polymorphism, according to large Mendelian randomization studies,^12,13^ it remains to be demonstrated in lower-limb atherosclerosis development. Attention should also be paid to the potential benefits of Lp(a)-lowering treatment for lower-limb vascular risk. To this point, few treatments are available for investigation. A prespecified analysis of the FOURIER (Further Cardiovascular Outcomes Research With PCSK9 Inhibition in Subjects With Elevated Risk) trial showed that PCSK9 (proprotein convertase subtilisin/kexin 9) inhibition with evolocumab significantly reduced the Lp(a) level by approximately 27% among patients who received treatment and decreased the risk of coronary heart disease, death, MI, or urgent revascularization by 23% in patients with Lp(a) levels higher than 15.4 mg/dL.^69^ Moreover, a recent phase 2 trial assessing hepatocyte-directed antisense oligonucleotide inhibiting apolipoprotein(a) production showed a dose-dependent reduction of Lp(a) levels among patients with established cardiovascular disease and elevated Lp(a) levels,^29^ but the benefits for cardiovascular outcomes are still unknown.

### Limitations

This study presents several limitations. First, it is a monocentric cohort analysis with a potential attrition bias given that some patients may have been admitted to another hospital for a qualifying major adverse limb event composite outcome, such as a lower-limb revascularization or major amputation. Thus, we underestimated the true incidence rate, which reinforced the power of the associations we could have found. Second, some medical information was extracted from a medicoadministrative database using *ICD-10* diagnosis codes. Consequently, some data were lacking that described patients’ arterial status more precisely, such as functional symptoms, ankle brachial index measurements, ultrasonography results, or radiological anatomic patterns of arterial lesions. In particular, we could not ensure the vascular nature of coded amputations. Moreover, *ICD-10* diagnosis codes might include inaccuracies or underestimation because they are coded for the French Hospital Discharge Database, a system initially used for hospital pricing. However, external validity of the methods comes from other published cardiovascular epidemiological studies on hospital inpatients in France.^70,71,72,73,74^ As a retrospective cohort analysis, this study might have residual confounding issues, and there is a possibility that unmeasured variables could explain the association between Lp(a) and major limb events. We tried to limit those biases by conducting an adjusted AFT regression analysis.

Third, this study covered 2 decades, during which endovascular interventional radiology made substantial progress, and its use spread widely.^75,76^ For this reason, we performed a sensitivity analysis on the primary outcome by considering a time effect variable, and we found that the sensitivity analysis results were consistent with those of the primary analysis.

## Conclusions

In this cohort study, Lp(a) level was associated with an increased risk of lower-limb artery revascularization or major amputation among a large cohort of unselected hospitalized patients. Lipoprotein(a) needs to be considered to improve not only the cardiovascular risk but also the lower-limb vascular risk assessment.

## Notes

Supplement. **eTable 1.** Sensitivity Analyses With the Primary Outcome Models, Adding Hypolipemiant Drug Status on Patients With Available Data **eTable 2.** Sensitivity Analyses With the Primary Outcome Models, Adding Time Effect Variable **eTable 3.** Sensitivity Analyses With the Primary Outcome Models, Adding PAD, History of Ischaemic Heart Disease and History of Stroke **eTable 4.** Baseline Characteristics According to Subgroups **eTable 5.** Accelerated Failure Time (AFT) Models With MALE as the Outcome Variable **eTable 6.** Accelerated Failure Time (AFT) Models With Major Amputation as the Outcome Variable **eTable 7.** Accelerated Failure Time (AFT) Models With Peripheral Endovascular Revascularization as the Outcome Variable **eTable 8.** Accelerated Failure Time (AFT) Models With Peripheral Surgical Revascularization as the Outcome Variable **eTable 9.** *P*-values Included in the Benjamini-Hochberg False Discovery Rate Approach **eFigure 1.** Frequency Distribution of Lipoprotein(a) [Lp(a)] in All Patients **eFigure 2.** Kaplan-Meier Survival Curves of the Cumulative Incidence of Major Amputation by Lp(a) Levels **eFigure 3.** Kaplan-Meier Survival Curves of the Cumulative Incidence of Peripheral Endovascular Revascularization by Lp(a) Levels **eFigure 4.** Kaplan-Meier Survival Curves of the Cumulative Incidence of Peripheral Surgical Revascularization by Lp(a) Levels **eFigure 5.** Distribution of Patients by Hospitalization Unit and Lp(a) Levels at Inclusion **eMethods 1.** List of ICD-10 Used for Comorbidities Definitions **eMethods 2.** List of CCAM (French Common Classification of Medical Procedures) Used for Outcomes Definitions

Supplement 2.
Data Sharing Statement
