# Supplementary material for: Association of Lipoprotein(a) Levels With Incidence of Major Adverse Limb Events
Source: JAMA Netw Open. 2022 Dec 8;5(12):e2245720. doi: 10.1001/jamanetworkopen.2022.45720 (PMC9856359; doi:10.1001/jamanetworkopen.2022.45720)
Supplement: Supplement. — eTable 1. Sensitivity Analyses With the Primary Outcome Models, Adding Hypolipemiant Drug Status on Patients With Available Data eTable 2. Sensitivity Analyses With the Primary Outcome Models, Adding Time Effect Variable eTable 3. Sensitivity Analyses With the Primary Outcome Models, Adding PAD, History of Ischaemic Heart Disease and History of Stroke eTable 4. Baseline Characteristics According to Subgroups eTable 5. Accelerated Failure Time (AFT) Models With MALE as the Outcome Variable eTable 6. Accelerated Failure Time (AFT) Models With Major Amputation as the Outcome Variable eTable 7. Accelerated Failure Time (AFT) Models With Peripheral Endovascular Revascularization as the Outcome Variable eTable 8. Accelerated Failure Time (AFT) Models With Peripheral Surgical Revascularization as the Outcome Variable eTable 9. P-values Included in the Benjamini-Hochberg False Discovery Rate Approach eFigure 1. Frequency Distribution of Lipoprotein(a) [Lp(a)] in All Patients eFigure 2. Kaplan-Meier Survival Curves of the Cumulative Incidence of Major Amputation by Lp(a) Levels eFigure 3. Kaplan-Meier Survival Curves of the Cumulative Incidence of Peripheral Endovascular Revascularization by Lp(a) Levels eFigure 4. Kaplan-Meier Survival Curves of the Cumulative Incidence of Peripheral Surgical Revascularization by Lp(a) Levels eFigure 5. Distribution of Patients by Hospitalization Unit and Lp(a) Levels at Inclusion eMethods 1. List of ICD-10 Used for Comorbidities Definitions eMethods 2. List of CCAM (French Common Classification of Medical Procedures) Used for Outcomes Definitions [file jamanetwopen-e2245720-s001.pdf]

# Supplemental Online Content

Guédon AF, De Freminville J-B, Mirault T, et al. Association of lipoprotein(a) levels with incidence of major adverse limb events. *JAMA Netw Open*. 2022;5(12):e2245720. doi:10.1001/jamanetworkopen.2022.45720

**eTable 1.** Sensitivity Analyses With the Primary Outcome Models, Adding Hypolipemiant Drug Status on Patients With Available Data

**eTable 2.** Sensitivity Analyses With the Primary Outcome Models, Adding Time Effect Variable

**eTable 3.** Sensitivity Analyses With the Primary Outcome Models, Adding PAD, History of Ischaemic Heart Disease and History of Stroke

**eTable 4.** Baseline Characteristics According to Subgroups

**eTable 5.** Accelerated Failure Time (AFT) Models With MALE as the Outcome Variable

**eTable 6.** Accelerated Failure Time (AFT) Models With Major Amputation as the Outcome Variable

**eTable 7.** Accelerated Failure Time (AFT) Models With Peripheral Endovascular Revascularization as the Outcome Variable

**eTable 8.** Accelerated Failure Time (AFT) Models With Peripheral Surgical Revascularization as the Outcome Variable

**eTable 9.** *P*-values Included in the Benjamini-Hochberg False Discovery Rate Approach

**eFigure 1.** Frequency Distribution of Lipoprotein(a) [Lp(a)] in All Patients

**eFigure 2.** Kaplan-Meier Survival Curves of the Cumulative Incidence of Major Amputation by Lp(a) Levels

**eFigure 3.** Kaplan-Meier Survival Curves of the Cumulative Incidence of Peripheral Endovascular Revascularization by Lp(a) Levels

**eFigure 4.** Kaplan-Meier Survival Curves of the Cumulative Incidence of Peripheral Surgical Revascularization by Lp(a) Levels

**eFigure 5.** Distribution of Patients by Hospitalization Unit and Lp(a) Levels at Inclusion

© 2022 Guédon AF et al. *JAMA Network Open.*

**eMethods 1.** List of ICD-10 Used for Comorbidities Definitions

**eMethods 2.** List of CCAM (French Common Classification of Medical Procedures) Used for Outcomes Definitions

This supplemental material has been provided by the authors to give readers additional information about their work.

**eTable 1.** Sensitivity Analyses With the Primary Outcome Models, Adding Hypolipemiant Drug Status on Patients With Available Data

| **MALE outcome AFT models - log(Lp(a)) adjusted with hypolipemiant drugs** | | | | |
|---|---|---|---|---|
| **Variable** | **Adjusted AFT Exponential Estimate** | **CI 95%** | **Uncorrected p-value** | **Benjamini - Hochberg corrected p-value** |
| Log[Lp(a)] | 0.69 | [0.53 ; 0.89] | 0.0043081 | 0,00710836 |
| Age, y | 0.94 | [0.92 ; 0.96] | 0.0000001 | 0,00000025 |
| Male sex | 0.42 | [0.22 ; 0.83] | 0.0117312 | 0,01738026 |
| Log(Serum creatinine) | 1.43 | [0.81 ; 2.53] | 0.2141561 | 0,23380689 |
| History of smoking (former or current) | 0.18 | [0.10 ; 0.34] | 0.0000002 | 0,00000050 |
| Diabetes mellitus | 0.10 | [0.05 ; 0.19] | 0.0000001 | 0,00000025 |
| LDL cholesterol | 2.34 | [1.72 ; 3.19] | 0.0000001 | 0,00000025 |
| Arterial hypertension | 1.05 | [0.54 ; 2.01] | 0.8900296 | 0,90838073 |
| Dialysis | 0.36 | [0.01 ; 13.22] | 0.5790493 | 0,60343032 |
| Hypolipemiant drugs | 0.05 | [0.02 ; 0.10] | 0.0000001 | 0,00000025 |
| **MALE outcome AFT models - Lp(a) threshold adjusted with hypolipemiant drugs** | | | | |
| **Variable** | **Adjusted AFT Exponential Estimate** | **CI 95%** | **Uncorrected p-value** | **Benjamini - Hochberg corrected p-value** |
| High Lp(a) [a] | 0.81 | [0.41 ; 1.59] | 0.5394637 | 0,56815858 |
| Very High Lp(a) [a] | 0.33 | [0.11 ; 0.94] | 0.0389911 | 0,05216377 |
| Age, y | 0.94 | [0.92 ; 0.96] | 0.0000001 | 0,00000025 |
| Male sex | 0.43 | [0.22 ; 0.84] | 0.0138744 | 0,01970963 |
| Log(Serum creatinine) | 1.37 | [0.78 ; 2.41] | 0.2721395 | 0,29284577 |
| History of smoking (former or current) | 0.19 | [0.10 ; 0.36] | 0.0000003 | 0,00000072 |
| Diabetes mellitus | 0.09 | [0.05 ; 0.19] | 0.0000001 | 0,00000025 |
| LDL cholesterol | 2.29 | [1.69 ; 3.12] | 0.0000001 | 0,00000025 |
| Arterial hypertension | 1.02 | [0.53 ; 1.97] | 0.9465362 | 0,94653620 |
| Dialysis | 0.40 | [0.01 ; 14.40] | 0.6127007 | 0,63184760 |
| Hypolipemiant drugs | 0.05 | [0.02 ; 0.10] | 0.0000001 | 0,00000025 |

[a]Compared with Normal Lp(a) as reference variable
AFT: Accelerated Failure Time ; CI: confidence interval LDL: low density lipoprotein ; Lp(a): Lipoprotein(a) ; MALE: Major Adverse Limb Events

© 2022 Guédon AF et al. *JAMA Network Open.*

**eTable 2.** Sensitivity Analyses With the Primary Outcome Models, Adding Time Effect Variable

| **MALE outcome AFT models - Log[Lp(a)] non adjusted with time effect** | | | |
|---|---|---|---|
| **Variable** | **Non adjusted AFT Exponential Estimate [CI95%]** | **Uncorrected p-value** | **Benjamini-Hochberg corrected p-value** |
| Log[Lp(a)] | 0.66 [0.53 ; 0.82] | 0.0001385 | 0,00027423 |
| Time effect | 19.41 [11.48 ; 32.82] | 0.0000001 | 0,00000025 |
| **MALE outcome AFT models - Lp(a) threshold non adjusted with time effect** | | | |
| **Variable** | **Non adjusted AFT Exponential Estimate [CI95%]** | **Uncorrected p-value** | **Benjamini-Hochberg corrected p-value** |
| High Lp(a) [a] | 0.55 [0.31 ; 0.99] | 0.0459982 | 0,06071762 |
| Very High Lp(a) [a] | 0.28 [0.12 ; 0.66] | 0.0038727 | 0,00661030 |
| Time effect | 19.30 [11.41 ; 32.67] | 0.0000001 | 0,00000025 |
| **MALE outcome AFT models - Log[Lp(a)] adjusted with time effect** | | | |
| **Variable** | **Adjusted AFT Exponential Estimate [CI95%]** | **Uncorrected p-value** | **Benjamini -Hochberg corrected p-value** |
| Log[Lp(a)] | 0.60 [0.49 ; 0.74] | 0.0000014 | 0,00000315 |
| Age, y | 0.91 [0.90 ; 0.93] | 0.0000001 | 0,00000025 |
| Male sex | 0.44 [0.25 ; 0.76] | 0.0030043 | 0,00521799 |
| Log(Serum creatinine) | 0.69 [0.40 ; 1.17] | 0.1664505 | 0,18725681 |
| History of smoking (former or current) | 0.19 [0.12 ; 0.32] | 0.0000001 | 0,00000025 |
| Diabetes mellitus | 0.07 [0.04 ; 0.12] | 0.0000001 | 0,00000025 |
| LDL cholesterol | 4.90 [3.71 ; 6.47] | 0.0000001 | 0,00000025 |
| Arterial hypertension | 1.90 [1.15 ; 3.14] | 0.0117624 | 0,01738026 |
| Dialysis | 0.08 [0.01 ; 1.00] | 0.0504431 | 0,06485541 |
| Time effect | 9.63 [5.89 ; 15.74] | 0.0000001 | 0,00000025 |
| **MALE outcome AFT models - Lp(a) threshold adjusted with time effect** | | | |
| **Variable** | **Adjusted AFT Exponential Estimate [CI95%]** | **Uncorrected p-value** | **Benjamini -Hochberg corrected p-value** |
| High Lp(a) [a] | 0.46 [0.26 ; 0.80] | 0.0063386 | 0,01012131 |
| Very High Lp(a) [a] | 0.23 [0.10 ; 0.54] | 0.0005987 | 0,00113983 |
| Age, y | 0.91 [0.90 ; 0.93] | 0.0000001 | 0,00000025 |
| Male sex | 0.45 [0.26 ; 0.78] | 0.0041064 | 0,00689040 |
| Log(Serum creatinine) | 0.66 [0.39 ; 1.13] | 0.1313049 | 0,15811600 |
| History of smoking (former or current) | 0.20 [0.12 ; 0.32] | 0.0000001 | 0,00000025 |
| Diabetes mellitus | 0.06 [0.04 ; 0.12] | 0.0000001 | 0,00000025 |
| LDL cholesterol | 4.81 [3.65 ; 6.35] | 0.0000001 | 0,00000025 |
| Arterial hypertension | 1.88[1.14 ; 3.10] | 0.0139361 | 0,01970963 |
| Dialysis | 0.08 [0.01 ; 0.98] | 0.0480577 | 0,06260148 |
| Time effect | 9.56 [5.84 ; 15.65] | 0.0000001 | 0,00000025 |

[a]Compared with Normal Lp(a) as reference variable
AFT: Accelerated Failure Time ; CI: confidence interval LDL: low density lipoprotein ; Lp(a): Lipoprotein(a) ; MALE: Major Adverse Limb Events

Time effect is defined as the binary variable inclusion time before or after the median inclusion time of follow up cohort, set to September 29, 2011

© 2022 Guédon AF et al. *JAMA Network Open.*

**eTable 3.** Sensitivity Analyses With the Primary Outcome Models, Adding PAD, History of Ischaemic Heart Disease and History of Stroke

| MALE outcome AFT models - Log[Lp(a)] adjusted with PAD | | | | |
|---|---|---|---|---|
| **Variable** | **Adjusted AFT Exponential Estimate** | **CI95%** | **Uncorrected p-value** | **Benjamini-Hochberg corrected p-value** |
| Log[Lp(a)] | 0.82 | [0.69 ; 0.97] | 0.0192676 | 0.02686609 |
| Age, y | 0.96 | [0.95 ; 0.98] | 0.0000044 | 0.00000947 |
| Male sex | 0.73 | [0.47 ; 1.15] | 0.1708286 | 0.19002282 |
| Log(Serum creatinine) | 0.76 | [0.49 ; 1.17] | 0.2149134 | 0.23380689 |
| History of smoking (former or current) | 0.59 | [0.38 ; 0.89] | 0.0126084 | 0.01835635 |
| Diabetes mellitus | 0.19 | [0.12 ; 0.31] | 0.0000001 | 0.00000025 |
| LDL cholesterol | 2.24 | [1.81 ; 2.77] | 0.0000001 | 0.00000025 |
| Arterial hypertension | 0.98 | [0.64 ; 1.50] | 0.9285879 | 0.93806329 |
| Dialysis | 0.17 | [0.02 ; 1.17] | 0.0721859 | 0.09162057 |
| PAD | 0.0003 | [0.0002 ; 0.0007] | 0.0000001 | 0.00000025 |
| | | | | |
| | | | | |
| **MALE outcome AFT models - Log[Lp(a)] adjusted with history of ischaemic heart disease** | | | | |
| **Variable** | **Adjusted AFT Exponential Estimate** | **CI95%** | **Uncorrected p-value** | **Benjamini-Hochberg corrected p-value** |
| Log[Lp(a)] | 0.59 | [0.47 ; 0.73] | 0.0000018 | 0.00000396 |
| Age, y | 0.91 | [0.89 ; 0.93] | 0.0000001 | 0.00000025 |
| Male sex | 0.52 | [0.29 ; 0.93] | 0.0279242 | 0.03839578 |
| Log(Serum creatinine) | 0.66 | [0.37 ; 1.16] | 0.1500958 | 0.17481746 |
| History of smoking (former or current) | 0.19 | [0.11 ; 0.33] | 0.0000001 | 0.00000025 |
| Diabetes mellitus | 0.05 | [0.03 ; 0.10] | 0.0000001 | 0.00000025 |

© 2022 Guédon AF et al. *JAMA Network Open.*

| | | | | |
|---|---|---|---|---|
| LDL cholesterol | 5.31 | [3.95 ; 7.12] | 0.0000001 | 0.00000025 |
| Arterial hypertension | 2.36 | [1.39 ; 4.00] | 0.0014438 | 0.00269691 |
| Dialysis | 0.13 | [0.01 ; 1.85] | 0.1325619 | 0.15811600 |
| History of ischaemic heart disease | 0.30 | [0.17 ; 0.54] | 0.0000740 | 0.00014951 |
| | | | | |
| | | | | |
| **MALE outcome AFT models - Log[Lp(a)] adjusted with history of stroke** | | | | |
| **Variable** | **Adjusted AFT Exponential Estimate** | **CI95%** | **Uncorrected p-value** | **Benjamini-Hochberg corrected p-value** |
| Log[Lp(a)] | 0.57 | [0.46 ; 0.71] | 0.0000004 | 0.00000092 |
| Age, y | 0.90 | [0.89 ; 0.92] | 0.0000001 | 0.00000025 |
| Male sex | 0.46 | [0.26 ; 0.81] | 0.0071492 | 0.01105892 |
| Log(Serum creatinine) | 0.66 | [0.37 ; 1.17] | 0.1531036 | 0.17624717 |
| History of smoking (former or current) | 0.17 | [0.01 ; 0.29] | 0.0000001 | 0.00000025 |
| Diabetes mellitus | 0.05 | [0.03 ; 0.09] | 0.0000001 | 0.00000025 |
| LDL cholesterol | 5.69 | [4.23 ; 7.63] | 0.0000001 | 0.00000025 |
| Arterial hypertension | 2.32 | [1.37 ; 3.93] | 0.0017542 | 0.00318960 |
| Dialysis | 0.13 | [0.01 ; 1.74] | 0.1223558 | 0.14954598 |
| History of stroke | 0.40 | [0.06 ; 2.59] | 0.3368463 | 0.35857832 |

AFT: Accelerated Failure Time ; CI: confidence interval LDL: low density lipoprotein ; Lp(a): Lipoprotein(a) ; MALE: Major Adverse Limb Events; PAD : Peripheral Artery Disease

© 2022 Guédon AF et al. *JAMA Network Open.*

# **eTable 4.** Baseline Characteristics According to Subgroups

| | **InDay hospitalization** | **Conventionnal hospitalization** | **Ischemic heart disease** | **PAD** |
|---|---|---|---|---|
| number | 13661 | 2852 | 2387 | 1139 |
| Age, y | 57.89 [49.29, 65.92] | 60.17 [46.56, 71.96] | 63.67 [56.36, 71.02] | 65.07 [55.14, 74.87] |
| Male sex, n (%) | 8042 (58.9) | 1732 ( 60.7) | 1842 ( 77.2) | 799 ( 70.1) |
| Peripheral artery disease, n (%) | 311 ( 2.3) | 828 ( 29.0) | 338 ( 14.2) | 1139 (100.0) |
| Ischemic heart disease, n (%) | 1753 (12.8) | 634 ( 22.2) | 2387 (100.0) | 338 ( 29.7) |
| History of stroke, n (%) | 58 ( 0.4) | 146 ( 5.1) | 57 ( 2.4) | 33 ( 2.9) |
| Arterial hypertension, n (%) | 7621 (55.8) | 1441 ( 50.5) | 1456 ( 61.0) | 612 ( 53.7) |
| Cigarette use, n (%) | | | | |
| *Never smoked* | 9368 (68.6) | 1981 ( 69.5) | 1353 ( 56.7) | 646 ( 56.7) |
| *Former smoker* | 2589 (19.0) | 181 ( 6.3) | 576 ( 24.1) | 146 ( 12.8) |
| *Current smoker* | 1704 (12.5) | 690 ( 24.2) | 458 ( 19.2) | 347 ( 30.5) |
| Dyslipidemia, n (%) | 8743 (64.0) | 1759 ( 61.7) | 1960 ( 82.1) | 835 ( 73.3) |
| Diabetes mellitus, n (%) | 1553 (11.4) | 593 ( 20.8) | 544 ( 22.8) | 320 ( 28.1) |
| Dialysis, n (%) | 51 ( 0.4) | 24 ( 0.8) | 10 ( 0.4) | 7 ( 0.6) |
| Lp(a), mg/dL | 24.00 [10.00, 58.00] | 29.00 [11.00, 65.00] | 28.00 [11.00, 69.00] | 30.00 [12.00, 70.50] |
| Lp(a) thresold, n (%) | | | | |
| *Normal* | 9720 (71.2) | 1901 ( 66.7) | 1548 ( 64.9) | 726 ( 63.7) |
| *High* | 3322 (24.3) | 749 ( 26.3) | 681 ( 28.5) | 313 ( 27.5) |
| *Very high* | 619 ( 4.5) | 202 ( 7.1) | 158 ( 6.6) | 100 ( 8.8) |
| LDL cholesterol, mmol/L | 3.14 [2.48, 3.85] | 2.64 [1.99, 3.37] | 2.56 [1.94, 3.37] | 2.42 [1.76, 3.16] |
| HDL cholesterol, mmol/L | 1.29 [1.06, 1.57] | 1.14 [0.92, 1.41] | 1.16 [0.95, 1.42] | 1.13 [0.91, 1.38] |
| Total cholesterol, mmol/L | 5.12 [4.37, 5.94] | 4.55 [3.74, 5.43] | 4.49 [3.74, 5.42] | 4.32 [3.53, 5.21] |
| Triglycerides, mmol/L | 1.17 [0.84, 1.70] | 1.35 [0.98, 1.90] | 1.32 [0.95, 1.84] | 1.37 [1.03, 1.89] |
| Serum creatinine, µmol/L | 80.00 [68.00, 95.00] | 83.00 [65.25, 115.00] | 84.00 [72.00, 101.00] | 83.00 [66.00, 110.00] |
| Measured GFR, mL/min/1.73 m2 | 80.00 [67.00, 92.00] | 76.00 [52.00, 98.00] | 77.00 [63.00, 90.00] | 75.00 [53.00, 97.00] |
| Proteinuria Ur, g/L | 0.12 [0.08, 0.18] | 0.12 [0.06, 0.33] | 0.12 [0.08, 0.19] | 0.12 [0.06, 0.26] |
| Conjugated bilirubin, µmol/L | 3.00 [2.00, 4.00] | 5.00 [4.00, 8.00] | 4.00 [3.00, 5.00] | 5.00 [4.00, 9.00] |
| Total bilirubin, µmol/L | 12.00 [9.00, 16.00] | 10.00 [8.00, 14.00] | 12.00 [9.00, 16.00] | 10.00 [7.00, 14.00] |
| Gamma-glutamyltransferase, U/L | 21.00 [13.00, 35.00] | 28.00 [16.00, 57.00] | 25.00 [15.00, 43.00] | 30.00 [18.00, 57.00] |
| Alkaline phosphatase, (median [IQR]) | 59.00 [49.00, 72.00] | 70.00 [56.00, 90.00] | 61.00 [51.00, 75.00] | 70.00 [57.00, 90.00] |
| Alanine aminotransferase, U/L | 21.00 [15.00, 30.00] | 19.00 [13.00, 30.00] | 22.00 [15.00, 33.00] | 18.00 [13.00, 28.00] |
| Aspartate aminotransferase, U/L | 21.00 [17.00, 26.00] | 22.00 [17.00, 29.00] | 22.00 [18.00, 28.00] | 21.00 [17.00, 28.00] |
| Serum albumin, g/L | 39.00 [36.90, 41.27] | 34.00 [28.00, 38.50] | 34.95 [29.00, 38.00] | 34.50 [29.00, 38.00] |
| Hypolipemiant drugs, n/total n (%) | | 1520 ( 53.3) | 471 ( 74.3) | 628 ( 75.8) |

HDL : high density lipoprotein ; LDL: low density lipoprotein ; Lp(a): Lipoprotein(a) ; PAD : Peripheral Artery Disease

*Data regarding hypolipemiant drugs were not available for InDay hospitalization patients.*

© 2022 Guédon AF et al. *JAMA Network Open.*

## **eTable 5.** Accelerated Failure Time (AFT) Models With MALE as the Outcome Variable

| **MALE outcome AFT models - Log[Lp(a)] non adjusted** | | | |
|---|---|---|---|
| **Variable** | **Non adjusted AFT Exponential Estimate [CI95%]** | **Uncorrected p-value** | **Benjamini-Hochberg corrected p-value** |
| Log[Lp(a)] | 0.61 [0.48 ; 0.76] | 0.000018 | 0,00003791 |
| **MALE outcome AFT models - Lp(a) threshold non adjusted** | | | |
| **Variable** | **Non adjusted AFT Exponential Estimate [CI95%]** | **Uncorrected p-value** | **Benjamini - Hochberg corrected p-value** |
| High Lp(a)[a] | 0.50 [0.27 ; 0.93] | 0.029354 | 0,03980885 |
| Very High Lp(a)[a] | 0.18 [0.07 ; 0.44] | 0.000229 | 0,00044453 |
| **MALE outcome AFT models - Log[Lp(a)] adjusted** | | | |
| **Variable** | **Adjusted AFT Exponential Estimate [CI95%]** | **Uncorrected p-value** | **Benjamini - Hochberg corrected p-value** |
| Log[Lp(a)] | 0.57 [0.46 ; 0.71] | 0.0000004 | 0,00000092 |
| Age, y | 0.90 [0.88 ; 0.92] | 0.0000001 | 0,00000025 |
| Male sex | 0.46 [0.26 ; 0.81] | 0.0071317 | 0,01105892 |
| Log(Serum creatinine) | 0.67 [0.38 ; 1.18] | 0.1640512 | 0,18667895 |
| History of smoking (former or current) | 0.17 [0.10 ; 0.29] | 0.0000001 | 0,00000025 |
| Diabetes mellitus | 0.05 [0.03 ; 0.09] | 0.0000001 | 0,00000025 |
| LDL cholesterol | 5.71 [4.25 ; 7.68] | 0.0000001 | 0,00000025 |
| Arterial hypertension | 2.32 [1.37 ; 3.93] | 0.0017720 | 0,00318960 |
| Dialysis | 0.12 [0.01 ; 1.72] | 0.1200630 | 0,14857796 |
| **MALE outcome AFT models - Lp(a) threshold adjusted** | | | |
| **Variable** | **Adjusted AFT Exponential Estimate [CI95%]** | **Uncorrected p-value** | **Benjamini - Hochberg corrected p-value** |
| High Lp(a)[a] | 0.43 [0.24 ; 0.78] | 0.0051709 | 0,00839212 |
| Very High Lp(a)[a] | 0.17 [0.07 ; 0.40] | 0.0000664 | 0,00013695 |
| Age, y | 0.90 [0.89 ; 0.92] | 0.0000001 | 0,00000025 |
| Male sex | 0.47 [0.26 ; 0.83] | 0.0089485 | 0,01362925 |
| Log(Serum creatinine) | 0.65 [0.37 ; 1.14] | 0.1345821 | 0,15861462 |
| History of smoking (former or current) | 0.17 [0.10 ; 0.29] | 0.0000001 | 0,00000025 |
| Diabetes mellitus | 0.05 [0.03 ; 0.09] | 0.0000001 | 0,00000025 |
| LDL cholesterol | 5.61 [4.18 ; 7.53] | 0.0000001 | 0,00000025 |
| Arterial hypertension | 2.29 [1.35 ; 3.88] | 0.0021335 | 0,00377172 |
| Dialysis | 0.12 [0.01 ; 1.69] | 0.1164028 | 0,14587186 |

[a]Compared with Normal Lp(a) as reference variable
AFT: Accelerated Failure Time; CI: confidence interval LDL: low density lipoprotein; Lp(a): Lipoprotein(a); MALE: Major Adverse Limb Events

© 2022 Guédon AF et al. *JAMA Network Open.*

## **eTable 6.** Accelerated Failure Time (AFT) Models With Major Amputation as the Outcome Variable

| **Major amputation outcome AFT models - Log[Lp(a)] non adjusted** | | | |
|---|---|---|---|
| **Variable** | **Non adjusted AFT Exponential Estimate** | **CI95%** | **Uncorrected p-value** |
| Log[Lp(a)] | 0.56 | [0.35 ; 0.91] | 0.0184829 |
| **Major amputation outcome AFT models - Lp(a) threshold non adjusted** | | | |
| **Variable** | **Non adjusted AFT Exponential Estimate** | **CI95%** | **Uncorrected p-value** |
| High Lp(a) [a] | 0.42 | [0.12 ; 1.41] | 0.1601924 |
| Very High Lp(a) [a] | 0.22 | [0.03 ; 1.96] | 0.1755513 |
| **Major amputation outcome AFT models - Log[Lp(a)] adjusted** | | | |
| **Variable** | **Adjusted AFT Exponential Estimate** | **CI95%** | **Uncorrected p-value** |
| Log[Lp(a)] | 0.62 | [0.40 ; 0.98] | 0.0406164 |
| Age, y | 0.95 | [0.91 ; 0.99] | 0.0101601 |
| Male sex | 0.30 | [0.08 ; 1.09] | 0.0673985 |
| Log(Serum creatinine) | 0.19 | [0.07 ; 0.48] | 0.0005706 |
| History of smoking (former or current) | 0.88 | [0.27 ; 2.81] | 0.8279230 |
| Diabetes mellitus | 0.02 | [0.00 ; 0.06] | 0.0000000 |
| LDL cholesterol | 5.98 | [3.04 ; 11.77] | 0.0000002 |
| Arterial hypertension | 4.56 | [1.47 ; 14.15] | 0.0085716 |
| Dialysis | 0.11 | [0.00 ; 4.28] | 0.2380730 |
| **Major amputation outcome AFT models - Lp(a) threshold adjusted** | | | |
| **Variable** | **Adjusted AFT Exponential Estimate** | **CI95%** | **Uncorrected p-value** |
| High Lp(a) [a] | 0.41 | [0.13 ; 1.32] | 0.1348754 |
| Very High Lp(a) [a] | 0.41 | [0.05 ; 3.40] | 0.4078284 |
| Age, y | 0.95 | [0.91 ; 0.99] | 0.0094476 |
| Male sex | 0.31 | [0.09 ; 1.13] | 0.0770335 |
| Log(Serum creatinine) | 0.18 | [0.07 ; 0.46] | 0.0004038 |
| History of smoking (former or current) | 0.88 | [0.28 ; 2.84] | 0.8372066 |
| Diabetes mellitus | 0.01 | [0.00 ; 0.05] | 0.0000000 |
| LDL cholesterol | 5.94 | [3.02 ; 11.69] | 0.0000002 |
| Arterial hypertension | 4.47 | [1.44 ; 13.88] | 0.0096805 |
| Dialysis | 0.11 | [0.00 ; 4.10] | 0.2282675 |

[a]Compared with Normal Lp(a) as reference variable
AFT: Accelerated Failure Time; CI: confidence interval LDL: low density lipoprotein; Lp(a): Lipoprotein(a); MALE: Major Adverse Limb Events

© 2022 Guédon AF et al. *JAMA Network Open.*

# **eTable 7.** Accelerated Failure Time (AFT) Models With Peripheral Endovascular Revascularization as the Outcome Variable

| **Endovascular revascularization outcome AFT models - Log[Lp(a)] non adjusted** | | | |
|---|---|---|---|
| **Variable** | **Non adjusted AFT Exponential Estimate** | **CI95%** | **Uncorrected p-value** |
| Log[Lp(a)] | 0.49 | [0.37 ; 0.65] | 0.0000007 |
| **Endovascular revascularization outcome AFT models - Lp(a) threshold non adjusted** | | | |
| **Variable** | **Non adjusted AFT Exponential Estimate** | **CI95%** | **Uncorrected p-value** |
| High Lp(a) [a] | 0.41 | [0.20 ; 0.84] | 0.0153689 |
| Very High Lp(a) [a] | 0.07 | [0.02 ; 0.24] | 0.0000191 |
| **Endovascular revascularization outcome AFT models - log(Lp(a)) adjusted** | | | |
| **Variable** | **Adjusted AFT Exponential Estimate** | **CI95%** | **Uncorrected p-value** |
| Log[Lp(a)] | 0.49 | [0.37 ; 0.63] | 0.0000001 |
| Age, y | 0.89 | [0.87 ; 0.91] | 0.0000001 |
| Male sex | 0.63 | [0.32 ; 1.23] | 0.1765692 |
| Log(Serum creatinine) | 0.39 | [0.20 ; 0.74] | 0.0041594 |
| History of smoking (former or current) | 0.17 | [0.09 ; 0.33] | 0.0000001 |
| Diabetes mellitus | 0.03 | [0.02 ; 0.07] | 0.0000001 |
| LDL cholesterol | 6.00 | [4.21 ; 8.56] | 0.0000001 |
| Arterial hypertension | 2.03 | [1.08 ; 3.81] | 0.0278617 |
| Dialysis | 0.31 | [0.01 ; 7.20] | 0.4662040 |
| **Endovascular revascularization outcome AFT models - Lp(a) threshold adjusted** | | | |
| **Variable** | **Adjusted AFT Exponential Estimate** | **CI95%** | **Uncorrected p-value** |
| High Lp(a) [a] | 0.36 | [0.18 ; 0.70] | 0.0027467 |
| Very High Lp(a) [a] | 0.09 | [0.03 ; 0.29] | 0.0000513 |
| Age, y | 0.89 | [0.87 ; 0.91] | 0.0000000 |
| Male sex | 0.65 | [0.33 ; 1.27] | 0.2051801 |
| Log(Serum creatinine) | 0.37 | [0.20 ; 0.72] | 0.0029516 |
| History of smoking (former or current) | 0.18 | [0.09 ; 0.34] | 0.0000001 |
| Diabetes mellitus | 0.03 | [0.02 ; 0.07] | 0.0000001 |
| LDL cholesterol | 5.81 | [4.08 ; 8.26] | 0.0000001 |
| Arterial hypertension | 2.00 | [1.06 ; 3.75] | 0.0317191 |
| Dialysis | 0.30 | [0.01 ; 6.86] | 0.4481727 |

[a]Compared with Normal Lp(a) as reference variable
AFT: Accelerated Failure Time; CI: confidence interval LDL: low density lipoprotein; Lp(a): Lipoprotein(a); MALE: Major Adverse Limb Events

© 2022 Guédon AF et al. *JAMA Network Open.*

**eTable 8.** Accelerated Failure Time (AFT) Models With Peripheral Surgical Revascularization as the Outcome Variable

| **Surgical revascularization outcome AFT models - Log[Lp(a)] non adjusted** | | | |
|---|---|---|---|
| **Variable** | **Non adjusted AFT Exponential Estimate** | **CI95%** | **Uncorrected p-value** |
| Log[Lp(a)] | 0.80 | [0.60 ; 1.07] | 0.1341378 |
| **Surgical revascularization outcome AFT models - Lp(a) threshold non adjusted** | | | |
| **Variable** | **Non adjusted AFT Exponential Estimate** | **CI95%** | **Uncorrected p-value** |
| High Lp(a) [a] | 0.74 | [0.34 ; 1.61] | 0.4446194 |
| Very High Lp(a) [a] | 0.45 | [0.11 ; 1.89] | 0.2747988 |
| **Surgical revascularization outcome AFT models - Log[Lp(a)] adjusted** | | | |
| **Variable** | **Adjusted AFT Exponential Estimate** | **CI95%** | **Uncorrected p-value** |
| Log[Lp(a)] | 0.72 | [0.54 ; 0.95] | 0.0222878 |
| Age, y | 0.93 | [0.91 ; 0.96] | 0.0000011 |
| Male sex | 0.30 | [0.13 ; 0.67] | 0.0034721 |
| Log(Serum creatinine) | 1.70 | [0.72 ; 4.01] | 0.2254132 |
| History of smoking (former or current) | 0.15 | [0.07 ; 0.30] | 0.0000002 |
| Diabetes mellitus | 0.24 | [0.11 ; 0.55] | 0.0007395 |
| LDL cholesterol | 4.24 | [2.85 ; 6.32] | 0.0000000 |
| Arterial hypertension | 1.68 | [0.84 ; 3.37] | 0.1447163 |
| Dialysis | 0.14 | [0.00 ; 5.72] | 0.2998081 |
| **Surgical revascularization outcome AFT models - Lp(a) threshold adjusted** | | | |
| **Variable** | **Adjusted AFT Exponential Estimate** | **CI95%** | **Uncorrected p-value** |
| High Lp(a) [a] | 0.61 | [0.28 ; 1.29] | 0.1951324 |
| Very High Lp(a) [a] | 0.35 | [0.09 ; 1.45] | 0.1481035 |
| Age, y | 0.93 | [0.91 ; 0.96] | 0.0000011 |
| Male sex | 0.31 | [0.14 ; 0.69] | 0.0040603 |
| Log(Serum creatinine) | 1.66 | [0.70 ; 3.92] | 0.2456297 |
| History of smoking (former or current) | 0.15 | [0.07 ; 0.31] | 0.0000002 |
| Diabetes mellitus | 0.24 | [0.11 ; 0.55] | 0.0006552 |
| LDL cholesterol | 4.20 | [2.83 ; 6.26] | 0.0000000 |
| Arterial hypertension | 1.67 | [0.83 ; 3.35] | 0.1517667 |
| Dialysis | 0.13 | [0.00 ; 5.44] | 0.2879857 |

[a]Compared with Normal Lp(a) as reference variable
AFT: Accelerated Failure Time; CI: confidence interval LDL: low density lipoprotein; Lp(a): Lipoprotein(a); MALE: Major Adverse Limb Events

© 2022 Guédon AF et al. *JAMA Network Open.*

**eTable 9.** *P*-values Included in the Benjamini-Hochberg False Discovery Rate Approach

| AFT model | Variable | Uncorrected p-value | Benjamini-Hochberg corrected p-value |
|---|---|---|---|
| Unadjusted AFT model with Log[Lp(a)] (outcome MALE) | Log[Lp(a)] | 0.000018 | 0,00003791 |
| Unadjusted AFT model with Lp(a) threshold (outcome MALE) | High Lp(a) [a] | 0.029354 | 0,03980885 |
| | Very High Lp(a) [a] | 0.000229 | 0,00044453 |
| Adjusted AFT model with Log[Lp(a)] (outcome MALE) | Log[Lp(a)] | 0.0000004 | 0,00000092 |
| | Age, y | 0.0000001 | 0,00000025 |
| | Male sex | 0.0071317 | 0,01105892 |
| | Log(Serum creatinine) | 0.1640512 | 0,18667895 |
| | History of smoking (former or current) | 0.0000001 | 0,00000025 |
| | Diabetes mellitus | 0.0000001 | 0,00000025 |
| | LDL cholesterol | 0.0000001 | 0,00000025 |
| | Arterial hypertension | 0.0017720 | 0,00318960 |
| | Dialysis | 0.1200630 | 0,14857796 |
| Adjusted AFT model with Lp(a) threshold (outcome MALE) | High Lp(a) [a] | 0.0051709 | 0,00839212 |
| | Very High Lp(a) [a] | 0.0000664 | 0,00013695 |
| | Age, y | 0.0000001 | 0,00000025 |
| | Male sex | 0.0089485 | 0,01362925 |
| | Log(Serum creatinine) | 0.1345821 | 0,15861462 |
| | History of smoking (former or current) | 0.0000001 | 0,00000025 |
| | Diabetes mellitus | 0.0000001 | 0,00000025 |
| | LDL cholesterol | 0.0000001 | 0,00000025 |
| | Arterial hypertension | 0.0021335 | 0,00377172 |
| | Dialysis | 0.1164028 | 0,14587186 |
| Unadjusted AFT model with Log[Lp(a)] (outcome MALE) with time effect | Log[Lp(a)] | 0.0001385 | 0,00027423 |
| | Time effect | 0.0000001 | 0,00000025 |
| Unadjusted AFT model with Lp(a) threshold (outcome MALE) with time effect | High Lp(a) [a] | 0.0459982 | 0,06071762 |
| | Very High Lp(a) [a] | 0.0038727 | 0,00661030 |
| | Time effect | 0.0000001 | 0,00000025 |
| Adjusted AFT model with Log[Lp(a)] (outcome MALE) with time effect | Log[Lp(a)] | 0.0000014 | 0,00000315 |
| | Age, y | 0.0000001 | 0,00000025 |
| | Male sex | 0.0030043 | 0,00521799 |
| | Log(Serum creatinine) | 0.1664505 | 0,18725681 |
| | History of smoking (former or current) | 0.0000001 | 0,00000025 |
| | Diabetes mellitus | 0.0000001 | 0,00000025 |
| | LDL cholesterol | 0.0000001 | 0,00000025 |

© 2022 Guédon AF et al. *JAMA Network Open.*

| | | | |
|---|---|---|---|
| | Arterial hypertension | 0.0117624 | 0,01738026 |
| | Dialysis | 0.0504431 | 0,06485541 |
| | Time effect | 0.0000001 | 0,00000025 |
| Adjusted AFT model with Lp(a) threshold (outcome MALE) with time effect | High Lp(a) [a] | 0.0063386 | 0,01012131 |
| | Very High Lp(a) [a] | 0.0005987 | 0,00113983 |
| | Age, y | 0.0000001 | 0,00000025 |
| | Male sex | 0.0041064 | 0,00689040 |
| | Log(Serum creatinine) | 0.1313049 | 0,15811600 |
| | History of smoking (former or current) | 0.0000001 | 0,00000025 |
| | Diabetes mellitus | 0.0000001 | 0,00000025 |
| | LDL cholesterol | 0.0000001 | 0,00000025 |
| | Arterial hypertension | 0.0139361 | 0,01970963 |
| | Dialysis | 0.0480577 | 0,06260148 |
| | Time effect | 0.0000001 | 0,00000025 |
| Adjusted AFT model with Log[Lp(a)] (outcome MALE) with hypolipemiant drug status | Log[Lp(a)] | 0.0043081 | 0,00710836 |
| | Age, y | 0.0000001 | 0,00000025 |
| | Male sex | 0.0117312 | 0,01738026 |
| | Log(Serum creatinine) | 0.2141561 | 0,23380689 |
| | History of smoking (former or current) | 0.0000002 | 0,00000050 |
| | Diabetes mellitus | 0.0000001 | 0,00000025 |
| | LDL cholesterol | 0.0000001 | 0,00000025 |
| | Arterial hypertension | 0.8900296 | 0,90838073 |
| | Dialysis | 0.5790493 | 0,60343032 |
| | Hypolipemiant drugs | 0.0000001 | 0,00000025 |
| Adjusted AFT model with Lp(a) threshold (outcome MALE) with hypolipemiant drug status | High Lp(a) [a] | 0.5394637 | 0,56815858 |
| | Very High Lp(a) [a] | 0.0389911 | 0,05216377 |
| | Age, y | 0.0000001 | 0,00000025 |
| | Male sex | 0.0138744 | 0,01970963 |
| | Log(Serum creatinine) | 0.2721395 | 0,29284577 |
| | History of smoking (former or current) | 0.0000003 | 0,00000072 |
| | Diabetes mellitus | 0.0000001 | 0,00000025 |
| | LDL cholesterol | 0.0000001 | 0,00000025 |
| | Arterial hypertension | 0.9465362 | 0,94653620 |
| | Dialysis | 0.6127007 | 0,63184760 |
| | Hypolipemiant drugs | 0.0000001 | 0,00000025 |
| Adjusted AFT model with Log[Lp(a)] (outcome MALE) with PAD status | Log[Lp(a)] | 0.0192676 | 0,02686609 |
| | Age, y | 0.0000044 | 0,00000947 |
| | Male sex | 0.1708286 | 0,19002282 |
| | Log(Serum creatinine) | 0.2149134 | 0,23380689 |

© 2022 Guédon AF et al. *JAMA Network Open.*

| | | | |
|---|---|---|---|
| | History of smoking (former or current) | 0.0126084 | 0,01835635 |
| | Diabetes mellitus | 0.0000001 | 0,00000025 |
| | LDL cholesterol | 0.0000001 | 0,00000025 |
| | Arterial hypertension | 0.9285879 | 0,93806329 |
| | Dialysis | 0.0721859 | 0,09162057 |
| | Peripheral arterial disease | 0.0000001 | 0,00000025 |
| Adjusted AFT model with Log[Lp(a)] (outcome MALE) with history of ischaemic heart disease | Log[Lp(a)] | 0.0000018 | 0,00000396 |
| | Age, y | 0.0000001 | 0,00000025 |
| | Male sex | 0.0279242 | 0,03839578 |
| | Log(Serum creatinine) | 0.1500958 | 0,17481746 |
| | History of smoking (former or current) | 0.0000001 | 0,00000025 |
| | Diabetes mellitus | 0.0000001 | 0,00000025 |
| | LDL cholesterol | 0.0000001 | 0,00000025 |
| | Arterial hypertension | 0.0014438 | 0,00269691 |
| | Dialysis | 0.1325619 | 0,15811600 |
| | History of ischaemic heart disease | 0.0000740 | 0,00014951 |
| Adjusted AFT model with Log[Lp(a)] (outcome MALE) with history of stroke | Log[Lp(a)] | 0.0000004 | 0,00000092 |
| | Age, y | 0.0000001 | 0,00000025 |
| | Male sex | 0.0071492 | 0,01105892 |
| | Log(Serum creatinine) | 0.1531036 | 0,17624717 |
| | History of smoking (former or current) | 0.0000001 | 0,00000025 |
| | Diabetes mellitus | 0.0000001 | 0,00000025 |
| | LDL cholesterol | 0.0000001 | 0,00000025 |
| | Arterial hypertension | 0.0017542 | 0,00318960 |
| | Dialysis | 0.1223558 | 0,14954598 |
| | History of stroke | 0.3368463 | 0,35857832 |

[a]Compared with Normal Lp(a) as reference variable
AFT: Accelerated Failure Time; CI: confidence interval LDL: low density lipoprotein; Lp(a): Lipoprotein(a); MALE: Major Adverse Limb Events; PAD : Peripheral Artery Disease

Time effect is defined as the binary variable inclusion time before or after the median inclusion time of follow up cohort, set to September 29, 2011

© 2022 Guédon AF et al. *JAMA Network Open.*

**eFigure 1.** Frequency Distribution of Lipoprotein(a) [Lp(a)] in All Patients

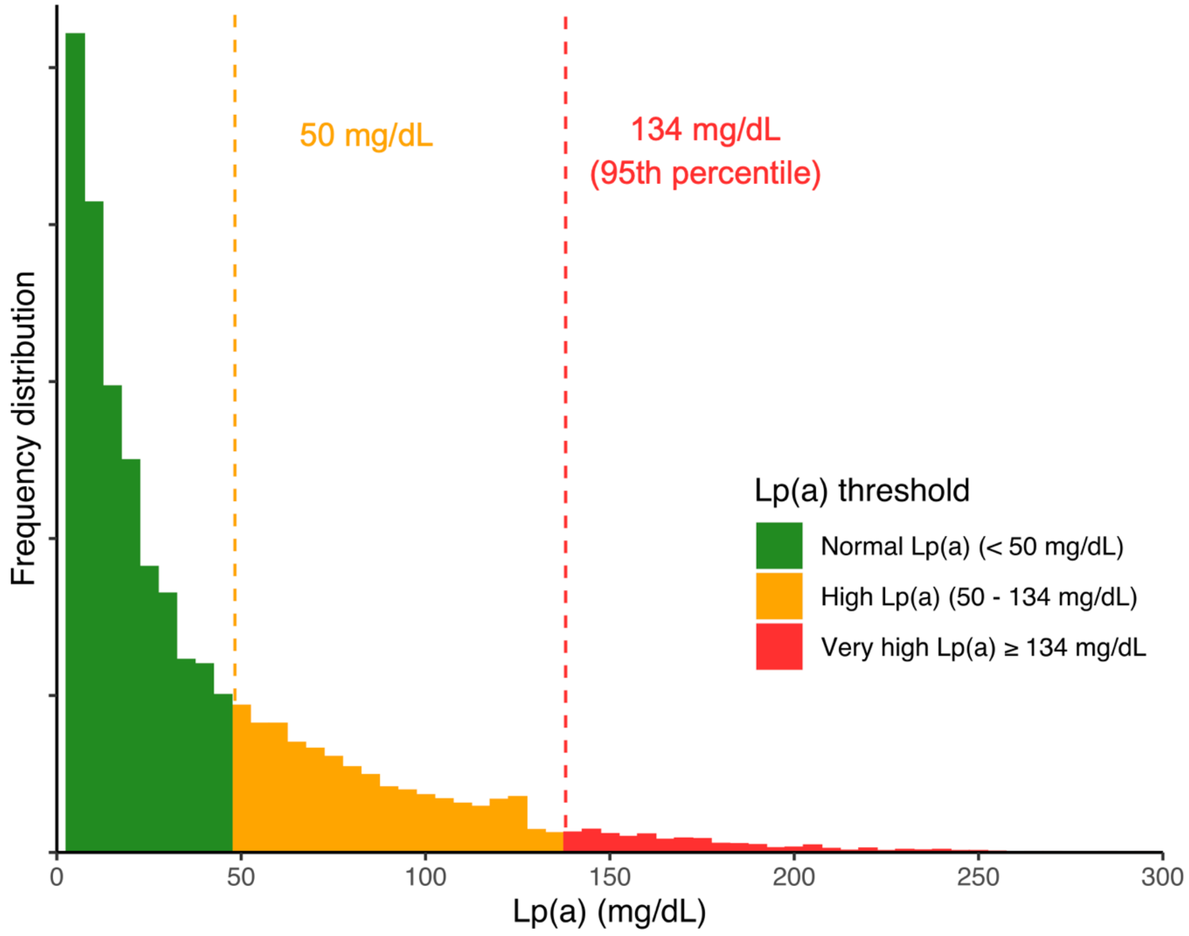


© 2022 Guédon AF et al. *JAMA Network Open.*

**eFigure 2.** Kaplan-Meier Survival Curves of the Cumulative Incidence of Major Amputation by Lp(a) Levels

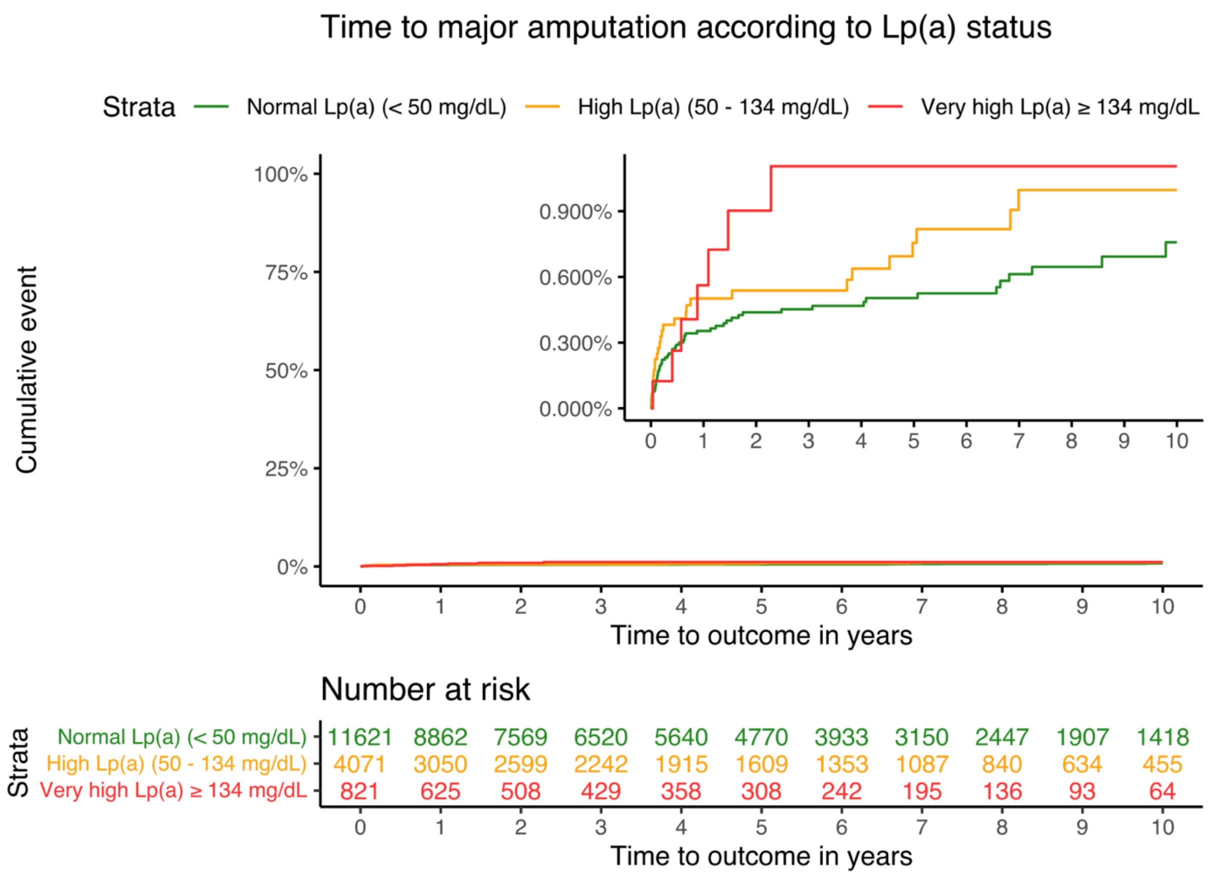


The inset shows the same data on an enlarged *y* axis.

© 2022 Guédon AF et al. *JAMA Network Open.*

**eFigure 3.** Kaplan-Meier Survival Curves of the Cumulative Incidence of Peripheral Endovascular Revascularization by Lp(a) Levels

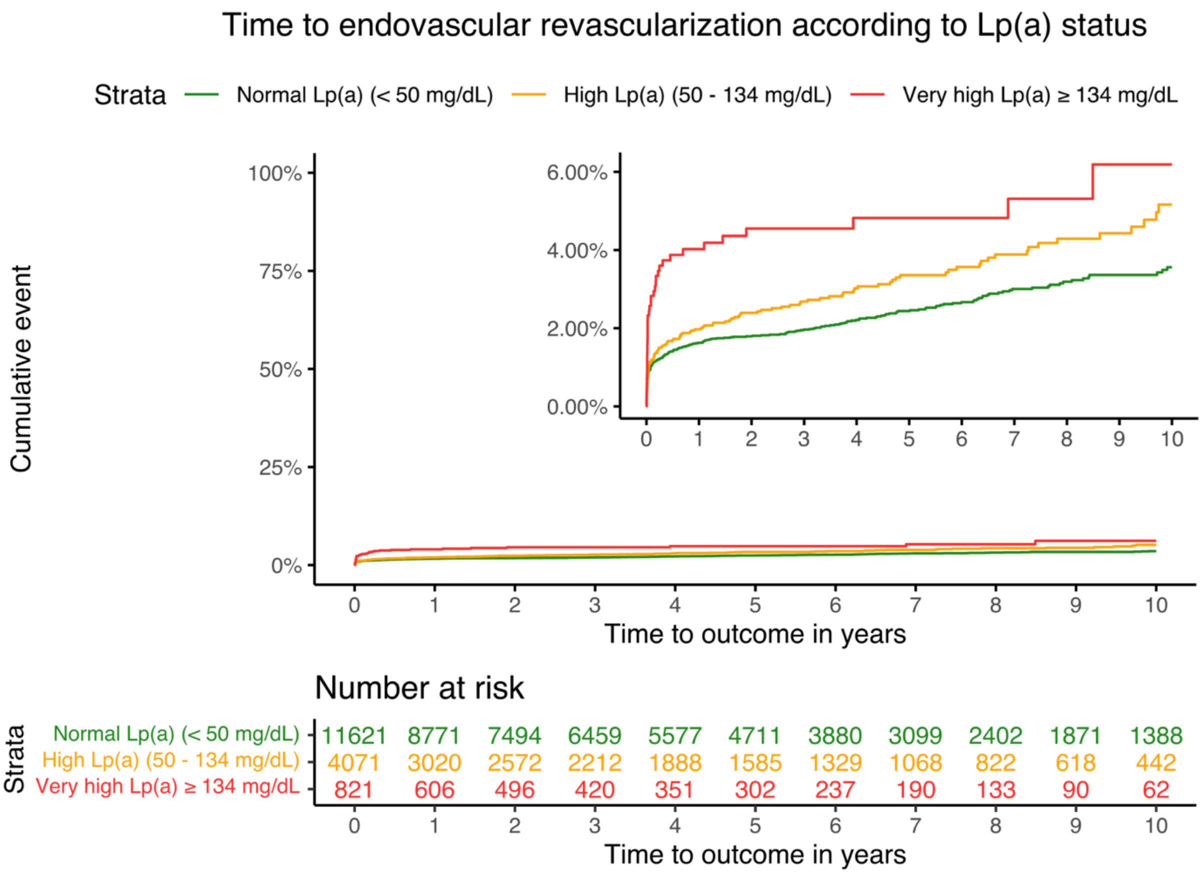


The inset shows the same data on an enlarged *y* axis.

© 2022 Guédon AF et al. *JAMA Network Open.*

**eFigure 4.** Kaplan-Meier Survival Curves of the Cumulative Incidence of Peripheral Surgical Revascularization by Lp(a) Levels

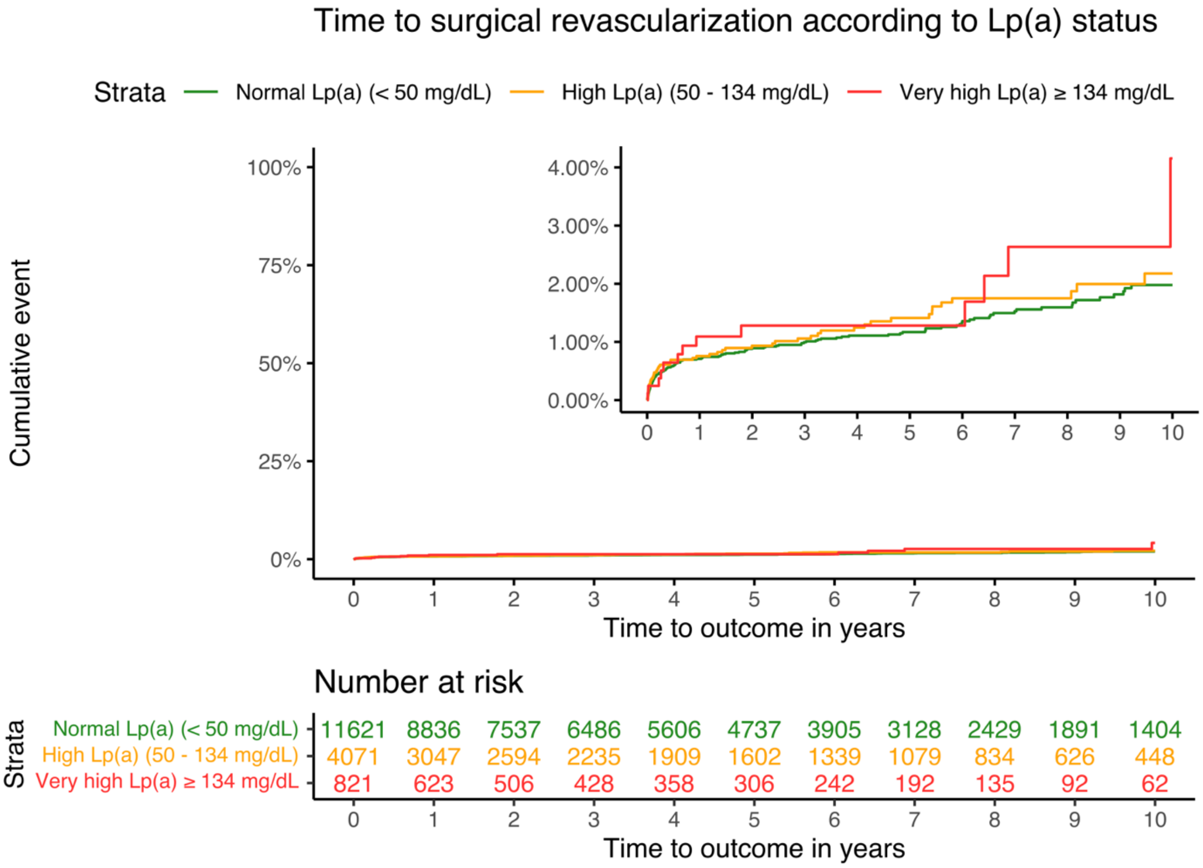


The inset shows the same data on an enlarged *y* axis.

© 2022 Guédon AF et al. *JAMA Network Open.*

**eFigure 5.** Distribution of Patients by Hospitalization Unit and Lp(a) Levels at Inclusion

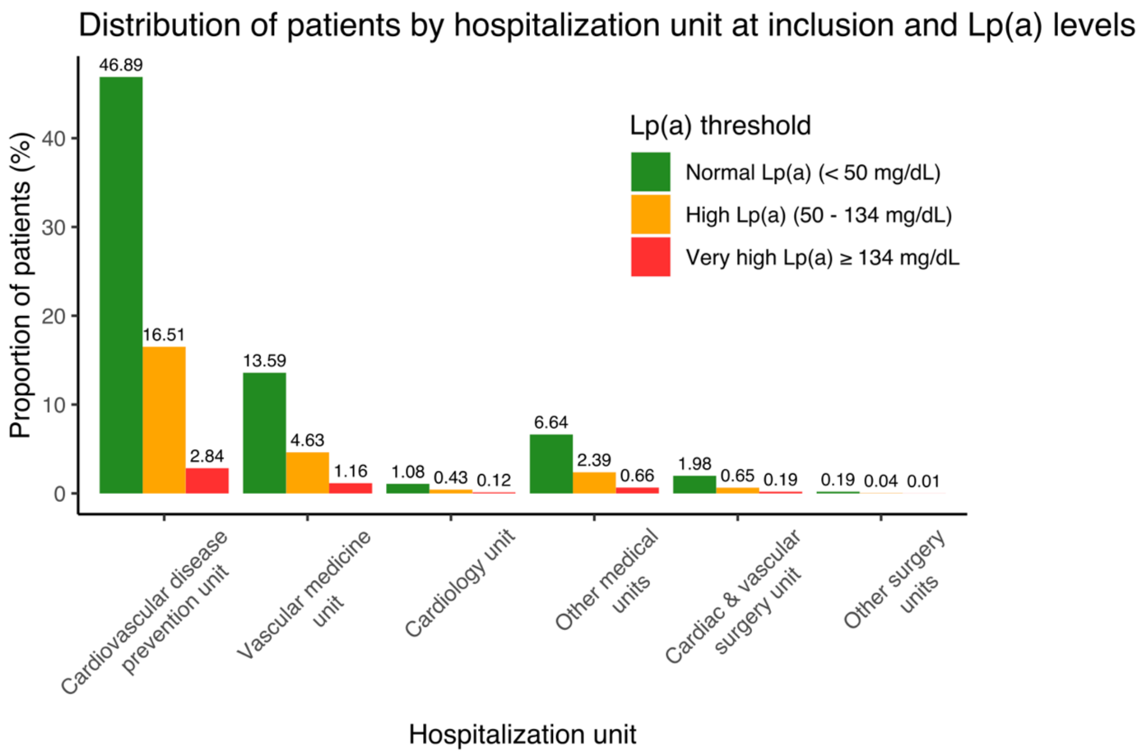


© 2022 Guédon AF et al. *JAMA Network Open.*

# **eMethods 1.** List of ICD-10 Used for Comorbidities Definitions

| **Variable** | **ICD-10 code** | **Label** |
|---|---|---|
| History of ischemic heart disease | I20 | Angina pectoris |
| History of ischemic heart disease | I200 | Unstable angina |
| History of ischemic heart disease | I201 | Angina pectoris with documented spasm |
| History of ischemic heart disease | I208 | Other forms of angina pectoris |
| History of ischemic heart disease | I209 | Angina pectoris, unspecified |
| History of ischemic heart disease | I21 | ST elevation (STEMI) and non-ST elevation (NSTEMI) myocardial infarction |
| History of ischemic heart disease | I210 | ST elevation (STEMI) myocardial infarction of anterior wall |
| History of ischemic heart disease | I2101 | ST elevation (STEMI) myocardial infarction involving left main coronary artery |
| History of ischemic heart disease | I2102 | ST elevation (STEMI) myocardial infarction involving left anterior descending coronary artery |
| History of ischemic heart disease | I2109 | ST elevation (STEMI) myocardial infarction involving other coronary artery of anterior wall |
| History of ischemic heart disease | I211 | ST elevation (STEMI) myocardial infarction of inferior wall |
| History of ischemic heart disease | I2111 | ST elevation (STEMI) myocardial infarction involving right coronary artery |
| History of ischemic heart disease | I2119 | ST elevation (STEMI) myocardial infarction involving other coronary artery of inferior wall |
| History of ischemic heart disease | I212 | ST elevation (STEMI) myocardial infarction of other sites |
| History of ischemic heart disease | I2121 | ST elevation (STEMI) myocardial infarction involving left circumflex coronary artery |
| History of ischemic heart disease | I2129 | ST elevation (STEMI) myocardial infarction involving other sites |
| History of ischemic heart disease | I213 | ST elevation (STEMI) myocardial infarction of unspecified site |
| History of ischemic heart disease | I214 | Non-ST elevation (NSTEMI) myocardial infarction |
| History of ischemic heart disease | I22 | Subsequent ST elevation (STEMI) and non-ST elevation (NSTEMI) myocardial infarction |
| History of ischemic heart disease | I220 | Subsequent ST elevation (STEMI) myocardial infarction of anterior wall |
| History of ischemic heart disease | I221 | Subsequent ST elevation (STEMI) myocardial infarction of inferior wall |
| History of ischemic heart disease | I222 | Subsequent non-ST elevation (NSTEMI) myocardial infarction |
| History of ischemic heart disease | I228 | Subsequent ST elevation (STEMI) myocardial infarction of other sites |
| History of ischemic heart disease | I229 | Subsequent ST elevation (STEMI) myocardial infarction of unspecified site |
| History of ischemic heart disease | I23 | Certain current complications following ST elevation (STEMI) and non-ST elevation (NSTEMI) myocardial infarction (within the 28 day period) |
| History of ischemic heart disease | I230 | Hemopericardium as current complication following acute myocardial infarction |
| History of ischemic heart disease | I231 | Atrial septal defect as current complication following acute myocardial infarction |
| History of ischemic heart disease | I232 | Ventricular septal defect as current complication following acute myocardial infarction |
| History of ischemic heart disease | I233 | Rupture of cardiac wall without hemopericardium as current complication following acute myocardial infarction |
| History of ischemic heart disease | I234 | Rupture of chordae tendineae as current complication following acute myocardial infarction |

© 2022 Guédon AF et al. *JAMA Network Open.*

| | | |
|---|---|---|
| History of ischemic heart disease | I235 | Rupture of papillary muscle as current complication following acute myocardial infarction |
| History of ischemic heart disease | I236 | Thrombosis of atrium, auricular appendage, and ventricle as current complications following acute myocardial infarction |
| History of ischemic heart disease | I237 | Postinfarction angina |
| History of ischemic heart disease | I238 | Other current complications following acute myocardial infarction |
| History of ischemic heart disease | I24 | Other acute ischemic heart diseases |
| History of ischemic heart disease | I240 | Acute coronary thrombosis not resulting in myocardial infarction |
| History of ischemic heart disease | I241 | Dressler's syndrome |
| History of ischemic heart disease | I248 | Other forms of acute ischemic heart disease |
| History of ischemic heart disease | I249 | Acute ischemic heart disease, unspecified |
| History of ischemic heart disease | I25 | Chronic ischemic heart disease |
| History of ischemic heart disease | I251 | Atherosclerotic heart disease of native coronary artery |
| History of ischemic heart disease | I2510 | Atherosclerotic heart disease of native coronary artery without angina pectoris |
| History of ischemic heart disease | I2511 | Atherosclerotic heart disease of native coronary artery with angina pectoris |
| History of ischemic heart disease | I25110 | Atherosclerotic heart disease of native coronary artery with unstable angina pectoris |
| History of ischemic heart disease | I25111 | Atherosclerotic heart disease of native coronary artery with angina pectoris with documented spasm |
| History of ischemic heart disease | I25118 | Atherosclerotic heart disease of native coronary artery with other forms of angina pectoris |
| History of ischemic heart disease | I25119 | Atherosclerotic heart disease of native coronary artery with unspecified angina pectoris |
| History of ischemic heart disease | I252 | Old myocardial infarction |
| History of ischemic heart disease | I253 | Aneurysm of heart |
| History of ischemic heart disease | I254 | Coronary artery aneurysm and dissection |
| History of ischemic heart disease | I2541 | Coronary artery aneurysm |
| History of ischemic heart disease | I2542 | Coronary artery dissection |
| History of ischemic heart disease | I255 | Ischemic cardiomyopathy |
| History of ischemic heart disease | I256 | Silent myocardial ischemia |
| History of ischemic heart disease | I257 | Atherosclerosis of coronary artery bypass graft(s) and coronary artery of transplanted heart with angina pectoris |
| History of ischemic heart disease | I2570 | Atherosclerosis of coronary artery bypass graft(s), unspecified, with angina pectoris |
| History of ischemic heart disease | I25700 | Atherosclerosis of coronary artery bypass graft(s), unspecified, with unstable angina pectoris |
| History of ischemic heart disease | I25701 | Atherosclerosis of coronary artery bypass graft(s), unspecified, with angina pectoris with documented spasm |
| History of ischemic heart disease | I25708 | Atherosclerosis of coronary artery bypass graft(s), unspecified, with other forms of angina pectoris |
| History of ischemic heart disease | I25709 | Atherosclerosis of coronary artery bypass graft(s), unspecified, with unspecified angina pectoris |
| History of ischemic heart disease | I2571 | Atherosclerosis of autologous vein coronary artery bypass graft(s) with angina pectoris |
| History of ischemic heart disease | I25710 | Atherosclerosis of autologous vein coronary artery bypass graft(s) with unstable angina pectoris |

© 2022 Guédon AF et al. *JAMA Network Open.*

| | | |
|---|---|---|
| History of ischemic heart disease | I25711 | Atherosclerosis of autologous vein coronary artery bypass graft(s) with angina pectoris with documented spasm |
| History of ischemic heart disease | I25718 | Atherosclerosis of autologous vein coronary artery bypass graft(s) with other forms of angina pectoris |
| History of ischemic heart disease | I25719 | Atherosclerosis of autologous vein coronary artery bypass graft(s) with unspecified angina pectoris |
| History of ischemic heart disease | I2572 | Atherosclerosis of autologous artery coronary artery bypass graft(s) with angina pectoris |
| History of ischemic heart disease | I25720 | Atherosclerosis of autologous artery coronary artery bypass graft(s) with unstable angina pectoris |
| History of ischemic heart disease | I25721 | Atherosclerosis of autologous artery coronary artery bypass graft(s) with angina pectoris with documented spasm |
| History of ischemic heart disease | I25728 | Atherosclerosis of autologous artery coronary artery bypass graft(s) with other forms of angina pectoris |
| History of ischemic heart disease | I25729 | Atherosclerosis of autologous artery coronary artery bypass graft(s) with unspecified angina pectoris |
| History of ischemic heart disease | I2573 | Atherosclerosis of nonautologous biological coronary artery bypass graft(s) with angina pectoris |
| History of ischemic heart disease | I25730 | Atherosclerosis of nonautologous biological coronary artery bypass graft(s) with unstable angina pectoris |
| History of ischemic heart disease | I25731 | Atherosclerosis of nonautologous biological coronary artery bypass graft(s) with angina pectoris with documented spasm |
| History of ischemic heart disease | I25738 | Atherosclerosis of nonautologous biological coronary artery bypass graft(s) with other forms of angina pectoris |
| History of ischemic heart disease | I25739 | Atherosclerosis of nonautologous biological coronary artery bypass graft(s) with unspecified angina pectoris |
| History of ischemic heart disease | I2575 | Atherosclerosis of native coronary artery of transplanted heart with angina pectoris |
| History of ischemic heart disease | I25750 | Atherosclerosis of native coronary artery of transplanted heart with unstable angina |
| History of ischemic heart disease | I25751 | Atherosclerosis of native coronary artery of transplanted heart with angina pectoris with documented spasm |
| History of ischemic heart disease | I25758 | Atherosclerosis of native coronary artery of transplanted heart with other forms of angina pectoris |
| History of ischemic heart disease | I25759 | Atherosclerosis of native coronary artery of transplanted heart with unspecified angina pectoris |
| History of ischemic heart disease | I2576 | Atherosclerosis of bypass graft of coronary artery of transplanted heart with angina pectoris |
| History of ischemic heart disease | I25760 | Atherosclerosis of bypass graft of coronary artery of transplanted heart with unstable angina |
| History of ischemic heart disease | I25761 | Atherosclerosis of bypass graft of coronary artery of transplanted heart with angina pectoris with documented spasm |
| History of ischemic heart disease | I25768 | Atherosclerosis of bypass graft of coronary artery of transplanted heart with other forms of angina pectoris |
| History of ischemic heart disease | I25769 | Atherosclerosis of bypass graft of coronary artery of transplanted heart with unspecified angina pectoris |
| History of ischemic heart disease | I2579 | Atherosclerosis of other coronary artery bypass graft(s) with angina pectoris |
| History of ischemic heart disease | I25790 | Atherosclerosis of other coronary artery bypass graft(s) with unstable angina pectoris |
| History of ischemic heart disease | I25791 | Atherosclerosis of other coronary artery bypass graft(s) with angina pectoris with documented spasm |
| History of ischemic heart disease | I25798 | Atherosclerosis of other coronary artery bypass graft(s) with other forms of angina pectoris |
| History of ischemic heart disease | I25799 | Atherosclerosis of other coronary artery bypass graft(s) with unspecified angina pectoris |
| History of ischemic heart disease | I258 | Other forms of chronic ischemic heart disease |
| History of ischemic heart disease | I2581 | Atherosclerosis of other coronary vessels without angina pectoris |
| History of ischemic heart disease | I25810 | Atherosclerosis of coronary artery bypass graft(s) without angina pectoris |
| History of ischemic heart disease | I25811 | Atherosclerosis of native coronary artery of transplanted heart without angina pectoris |

© 2022 Guédon AF et al. *JAMA Network Open.*

| | | |
|---|---|---|
| History of ischemic heart disease | I25812 | Atherosclerosis of bypass graft of coronary artery of transplanted heart without angina pectoris |
| History of ischemic heart disease | I2582 | Chronic total occlusion of coronary artery |
| History of ischemic heart disease | I2583 | Coronary atherosclerosis due to lipid rich plaque |
| History of ischemic heart disease | I2584 | Coronary atherosclerosis due to calcified coronary lesion |
| History of ischemic heart disease | I2589 | Other forms of chronic ischemic heart disease |
| History of ischemic heart disease | I259 | Chronic ischemic heart disease, unspecified |
| Peripheral artery disease | I702 | Atherosclerosis of native arteries of the extremities |
| Peripheral artery disease | I7020 | Unspecified atherosclerosis of native arteries of extremities |
| Peripheral artery disease | I7021 | Atherosclerosis of native arteries of extremities with intermittent claudication |
| Peripheral artery disease | I743 | Embolism and thrombosis of arteries of the lower extremities |
| History of stroke | I60 | Nontraumatic subarachnoid hemorrhage |
| History of stroke | I600 | Nontraumatic subarachnoid hemorrhage from carotid siphon and bifurcation |
| History of stroke | I6000 | Nontraumatic subarachnoid hemorrhage from unspecified carotid siphon and bifurcation |
| History of stroke | I6001 | Nontraumatic subarachnoid hemorrhage from right carotid siphon and bifurcation |
| History of stroke | I6002 | Nontraumatic subarachnoid hemorrhage from left carotid siphon and bifurcation |
| History of stroke | I601 | Nontraumatic subarachnoid hemorrhage from middle cerebral artery |
| History of stroke | I6010 | Nontraumatic subarachnoid hemorrhage from unspecified middle cerebral artery |
| History of stroke | I6011 | Nontraumatic subarachnoid hemorrhage from right middle cerebral artery |
| History of stroke | I6012 | Nontraumatic subarachnoid hemorrhage from left middle cerebral artery |
| History of stroke | I602 | Nontraumatic subarachnoid hemorrhage from anterior communicating artery |
| History of stroke | I6020 | Nontraumatic subarachnoid hemorrhage from unspecified anterior communicating artery |
| History of stroke | I6021 | Nontraumatic subarachnoid hemorrhage from right anterior communicating artery |
| History of stroke | I6022 | Nontraumatic subarachnoid hemorrhage from left anterior communicating artery |
| History of stroke | I603 | Nontraumatic subarachnoid hemorrhage from posterior communicating artery |
| History of stroke | I6030 | Nontraumatic subarachnoid hemorrhage from unspecified posterior communicating artery |
| History of stroke | I6031 | Nontraumatic subarachnoid hemorrhage from right posterior communicating artery |
| History of stroke | I6032 | Nontraumatic subarachnoid hemorrhage from left posterior communicating artery |
| History of stroke | I604 | Nontraumatic subarachnoid hemorrhage from basilar artery |
| History of stroke | I605 | Nontraumatic subarachnoid hemorrhage from vertebral artery |
| History of stroke | I6050 | Nontraumatic subarachnoid hemorrhage from unspecified vertebral artery |
| History of stroke | I6051 | Nontraumatic subarachnoid hemorrhage from right vertebral artery |
| History of stroke | I6052 | Nontraumatic subarachnoid hemorrhage from left vertebral artery |
| History of stroke | I606 | Nontraumatic subarachnoid hemorrhage from other intracranial arteries |
| History of stroke | I607 | Nontraumatic subarachnoid hemorrhage from unspecified intracranial artery |
| History of stroke | I608 | Other nontraumatic subarachnoid hemorrhage |
| History of stroke | I609 | Nontraumatic subarachnoid hemorrhage, unspecified |
| History of stroke | I61 | Nontraumatic intracerebral hemorrhage |
| History of stroke | I610 | Nontraumatic intracerebral hemorrhage in hemisphere, subcortical |
| History of stroke | I611 | Nontraumatic intracerebral hemorrhage in hemisphere, cortical |
| History of stroke | I612 | Nontraumatic intracerebral hemorrhage in hemisphere, unspecified |
| History of stroke | I613 | Nontraumatic intracerebral hemorrhage in brain stem |
| History of stroke | I614 | Nontraumatic intracerebral hemorrhage in cerebellum |
| History of stroke | I615 | Nontraumatic intracerebral hemorrhage, intraventricular |

© 2022 Guédon AF et al. *JAMA Network Open.*

| | | |
|---|---|---|
| History of stroke | I616 | Nontraumatic intracerebral hemorrhage, multiple localized |
| History of stroke | I618 | Other nontraumatic intracerebral hemorrhage |
| History of stroke | I619 | Nontraumatic intracerebral hemorrhage, unspecified |
| History of stroke | I62 | Other and unspecified nontraumatic intracranial hemorrhage |
| History of stroke | I620 | Nontraumatic subdural hemorrhage |
| History of stroke | I6200 | Nontraumatic subdural hemorrhage, unspecified |
| History of stroke | I6201 | Nontraumatic acute subdural hemorrhage |
| History of stroke | I6202 | Nontraumatic subacute subdural hemorrhage |
| History of stroke | I6203 | Nontraumatic chronic subdural hemorrhage |
| History of stroke | I621 | Nontraumatic extradural hemorrhage |
| History of stroke | I629 | Nontraumatic intracranial hemorrhage, unspecified |
| History of stroke | I63 | Cerebral infarction |
| History of stroke | I630 | Cerebral infarction due to thrombosis of precerebral arteries |
| History of stroke | I6300 | Cerebral infarction due to thrombosis of unspecified precerebral artery |
| History of stroke | I6301 | Cerebral infarction due to thrombosis of vertebral artery |
| History of stroke | I63011 | Cerebral infarction due to thrombosis of right vertebral artery |
| History of stroke | I63012 | Cerebral infarction due to thrombosis of left vertebral artery |
| History of stroke | I63019 | Cerebral infarction due to thrombosis of unspecified vertebral artery |
| History of stroke | I6302 | Cerebral infarction due to thrombosis of basilar artery |
| History of stroke | I6303 | Cerebral infarction due to thrombosis of carotid artery |
| History of stroke | I63031 | Cerebral infarction due to thrombosis of right carotid artery |
| History of stroke | I63032 | Cerebral infarction due to thrombosis of left carotid artery |
| History of stroke | I63039 | Cerebral infarction due to thrombosis of unspecified carotid artery |
| History of stroke | I6309 | Cerebral infarction due to thrombosis of other precerebral artery |
| History of stroke | I631 | Cerebral infarction due to embolism of precerebral arteries |
| History of stroke | I6310 | Cerebral infarction due to embolism of unspecified precerebral artery |
| History of stroke | I6311 | Cerebral infarction due to embolism of vertebral artery |
| History of stroke | I63111 | Cerebral infarction due to embolism of right vertebral artery |
| History of stroke | I63112 | Cerebral infarction due to embolism of left vertebral artery |
| History of stroke | I63119 | Cerebral infarction due to embolism of unspecified vertebral artery |
| History of stroke | I6312 | Cerebral infarction due to embolism of basilar artery |
| History of stroke | I6313 | Cerebral infarction due to embolism of carotid artery |
| History of stroke | I63131 | Cerebral infarction due to embolism of right carotid artery |
| History of stroke | I63132 | Cerebral infarction due to embolism of left carotid artery |
| History of stroke | I63139 | Cerebral infarction due to embolism of unspecified carotid artery |
| History of stroke | I6319 | Cerebral infarction due to embolism of other precerebral artery |
| History of stroke | I632 | Cerebral infarction due to unspecified occlusion or stenosis of precerebral arteries |
| History of stroke | I6320 | Cerebral infarction due to unspecified occlusion or stenosis of unspecified precerebral arteries |
| History of stroke | I6321 | Cerebral infarction due to unspecified occlusion or stenosis of vertebral arteries |
| History of stroke | I63211 | Cerebral infarction due to unspecified occlusion or stenosis of right vertebral arteries |
| History of stroke | I63212 | Cerebral infarction due to unspecified occlusion or stenosis of left vertebral arteries |
| History of stroke | I63219 | Cerebral infarction due to unspecified occlusion or stenosis of unspecified vertebral arteries |
| History of stroke | I6322 | Cerebral infarction due to unspecified occlusion or stenosis of basilar arteries |
| History of stroke | I6323 | Cerebral infarction due to unspecified occlusion or stenosis of carotid arteries |
| History of stroke | I63231 | Cerebral infarction due to unspecified occlusion or stenosis of right carotid arteries |
| History of stroke | I63232 | Cerebral infarction due to unspecified occlusion or stenosis of left carotid arteries |
| History of stroke | I63239 | Cerebral infarction due to unspecified occlusion or stenosis of unspecified carotid arteries |
| History of stroke | I6329 | Cerebral infarction due to unspecified occlusion or stenosis of other precerebral arteries |

© 2022 Guédon AF et al. *JAMA Network Open.*

| | | |
|---|---|---|
| History of stroke | I633 | Cerebral infarction due to thrombosis of cerebral arteries |
| History of stroke | I6330 | Cerebral infarction due to thrombosis of unspecified cerebral artery |
| History of stroke | I6331 | Cerebral infarction due to thrombosis of middle cerebral artery |
| History of stroke | I63311 | Cerebral infarction due to thrombosis of right middle cerebral artery |
| History of stroke | I63312 | Cerebral infarction due to thrombosis of left middle cerebral artery |
| History of stroke | I63319 | Cerebral infarction due to thrombosis of unspecified middle cerebral artery |
| History of stroke | I6332 | Cerebral infarction due to thrombosis of anterior cerebral artery |
| History of stroke | I63321 | Cerebral infarction due to thrombosis of right anterior cerebral artery |
| History of stroke | I63322 | Cerebral infarction due to thrombosis of left anterior cerebral artery |
| History of stroke | I63329 | Cerebral infarction due to thrombosis of unspecified anterior cerebral artery |
| History of stroke | I6333 | Cerebral infarction due to thrombosis of posterior cerebral artery |
| History of stroke | I63331 | Cerebral infarction due to thrombosis of right posterior cerebral artery |
| History of stroke | I63332 | Cerebral infarction due to thrombosis of left posterior cerebral artery |
| History of stroke | I63339 | Cerebral infarction due to thrombosis of unspecified posterior cerebral artery |
| History of stroke | I6334 | Cerebral infarction due to thrombosis of cerebellar artery |
| History of stroke | I63341 | Cerebral infarction due to thrombosis of right cerebellar artery |
| History of stroke | I63342 | Cerebral infarction due to thrombosis of left cerebellar artery |
| History of stroke | I63349 | Cerebral infarction due to thrombosis of unspecified cerebellar artery |
| History of stroke | I6339 | Cerebral infarction due to thrombosis of other cerebral artery |
| History of stroke | I634 | Cerebral infarction due to embolism of cerebral arteries |
| History of stroke | I6340 | Cerebral infarction due to embolism of unspecified cerebral artery |
| History of stroke | I6341 | Cerebral infarction due to embolism of middle cerebral artery |
| History of stroke | I63411 | Cerebral infarction due to embolism of right middle cerebral artery |
| History of stroke | I63412 | Cerebral infarction due to embolism of left middle cerebral artery |
| History of stroke | I63419 | Cerebral infarction due to embolism of unspecified middle cerebral artery |
| History of stroke | I6342 | Cerebral infarction due to embolism of anterior cerebral artery |
| History of stroke | I63421 | Cerebral infarction due to embolism of right anterior cerebral artery |
| History of stroke | I63422 | Cerebral infarction due to embolism of left anterior cerebral artery |
| History of stroke | I63429 | Cerebral infarction due to embolism of unspecified anterior cerebral artery |
| History of stroke | I6343 | Cerebral infarction due to embolism of posterior cerebral artery |
| History of stroke | I63431 | Cerebral infarction due to embolism of right posterior cerebral artery |
| History of stroke | I63432 | Cerebral infarction due to embolism of left posterior cerebral artery |
| History of stroke | I63439 | Cerebral infarction due to embolism of unspecified posterior cerebral artery |
| History of stroke | I6344 | Cerebral infarction due to embolism of cerebellar artery |
| History of stroke | I63441 | Cerebral infarction due to embolism of right cerebellar artery |
| History of stroke | I63442 | Cerebral infarction due to embolism of left cerebellar artery |
| History of stroke | I63449 | Cerebral infarction due to embolism of unspecified cerebellar artery |
| History of stroke | I6349 | Cerebral infarction due to embolism of other cerebral artery |
| History of stroke | I635 | Cerebral infarction due to unspecified occlusion or stenosis of cerebral arteries |
| History of stroke | I6350 | Cerebral infarction due to unspecified occlusion or stenosis of unspecified cerebral artery |
| History of stroke | I6351 | Cerebral infarction due to unspecified occlusion or stenosis of middle cerebral artery |
| History of stroke | I63511 | Cerebral infarction due to unspecified occlusion or stenosis of right middle cerebral artery |
| History of stroke | I63512 | Cerebral infarction due to unspecified occlusion or stenosis of left middle cerebral artery |
| History of stroke | I63519 | Cerebral infarction due to unspecified occlusion or stenosis of unspecified middle cerebral artery |
| History of stroke | I6352 | Cerebral infarction due to unspecified occlusion or stenosis of anterior cerebral artery |
| History of stroke | I63521 | Cerebral infarction due to unspecified occlusion or stenosis of right anterior cerebral artery |
| History of stroke | I63522 | Cerebral infarction due to unspecified occlusion or stenosis of left anterior cerebral artery |

© 2022 Guédon AF et al. *JAMA Network Open.*

| | | |
|---|---|---|
| History of stroke | I63529 | Cerebral infarction due to unspecified occlusion or stenosis of unspecified anterior cerebral artery |
| History of stroke | I6353 | Cerebral infarction due to unspecified occlusion or stenosis of posterior cerebral artery |
| History of stroke | I63531 | Cerebral infarction due to unspecified occlusion or stenosis of right posterior cerebral artery |
| History of stroke | I63532 | Cerebral infarction due to unspecified occlusion or stenosis of left posterior cerebral artery |
| History of stroke | I63539 | Cerebral infarction due to unspecified occlusion or stenosis of unspecified posterior cerebral artery |
| History of stroke | I6354 | Cerebral infarction due to unspecified occlusion or stenosis of cerebellar artery |
| History of stroke | I63541 | Cerebral infarction due to unspecified occlusion or stenosis of right cerebellar artery |
| History of stroke | I63542 | Cerebral infarction due to unspecified occlusion or stenosis of left cerebellar artery |
| History of stroke | I63549 | Cerebral infarction due to unspecified occlusion or stenosis of unspecified cerebellar artery |
| History of stroke | I6359 | Cerebral infarction due to unspecified occlusion or stenosis of other cerebral artery |
| History of stroke | I636 | Cerebral infarction due to cerebral venous thrombosis, nonpyogenic |
| History of stroke | I638 | Other cerebral infarction |
| History of stroke | I639 | Cerebral infarction, unspecified |
| History of stroke | I69 | Sequelae of cerebrovascular disease |
| History of stroke | I690 | Sequelae of nontraumatic subarachnoid hemorrhage |
| History of stroke | I6900 | Unspecified sequelae of nontraumatic subarachnoid hemorrhage |
| History of stroke | I6901 | Cognitive deficits following nontraumatic subarachnoid hemorrhage |
| History of stroke | I6902 | Speech and language deficits following nontraumatic subarachnoid hemorrhage |
| History of stroke | I69020 | Aphasia following nontraumatic subarachnoid hemorrhage |
| History of stroke | I69021 | Dysphasia following nontraumatic subarachnoid hemorrhage |
| History of stroke | I69022 | Dysarthria following nontraumatic subarachnoid hemorrhage |
| History of stroke | I69023 | Fluency disorder following nontraumatic subarachnoid hemorrhage |
| History of stroke | I69028 | Other speech and language deficits following nontraumatic subarachnoid hemorrhage |
| History of stroke | I6903 | Monoplegia of upper limb following nontraumatic subarachnoid hemorrhage |
| History of stroke | I69031 | Monoplegia of upper limb following nontraumatic subarachnoid hemorrhage affecting right dominant side |
| History of stroke | I69032 | Monoplegia of upper limb following nontraumatic subarachnoid hemorrhage affecting left dominant side |
| History of stroke | I69033 | Monoplegia of upper limb following nontraumatic subarachnoid hemorrhage affecting right non-dominant side |
| History of stroke | I69034 | Monoplegia of upper limb following nontraumatic subarachnoid hemorrhage affecting left non-dominant side |
| History of stroke | I69039 | Monoplegia of upper limb following nontraumatic subarachnoid hemorrhage affecting unspecified side |
| History of stroke | I6904 | Monoplegia of lower limb following nontraumatic subarachnoid hemorrhage |
| History of stroke | I69041 | Monoplegia of lower limb following nontraumatic subarachnoid hemorrhage affecting right dominant side |
| History of stroke | I69042 | Monoplegia of lower limb following nontraumatic subarachnoid hemorrhage affecting left dominant side |
| History of stroke | I69043 | Monoplegia of lower limb following nontraumatic subarachnoid hemorrhage affecting right non-dominant side |
| History of stroke | I69044 | Monoplegia of lower limb following nontraumatic subarachnoid hemorrhage affecting left non-dominant side |
| History of stroke | I69049 | Monoplegia of lower limb following nontraumatic subarachnoid hemorrhage affecting unspecified side |
| History of stroke | I6905 | Hemiplegia and hemiparesis following nontraumatic subarachnoid hemorrhage |
| History of stroke | I69051 | Hemiplegia and hemiparesis following nontraumatic subarachnoid hemorrhage affecting right dominant side |
| History of stroke | I69052 | Hemiplegia and hemiparesis following nontraumatic subarachnoid hemorrhage affecting left dominant side |
| History of stroke | I69053 | Hemiplegia and hemiparesis following nontraumatic subarachnoid hemorrhage affecting right non-dominant side |

© 2022 Guédon AF et al. *JAMA Network Open.*

| | | |
|---|---|---|
| History of stroke | I69054 | Hemiplegia and hemiparesis following nontraumatic subarachnoid hemorrhage affecting left non-dominant side |
| History of stroke | I69059 | Hemiplegia and hemiparesis following nontraumatic subarachnoid hemorrhage affecting unspecified side |
| History of stroke | I6906 | Other paralytic syndrome following nontraumatic subarachnoid hemorrhage |
| History of stroke | I69061 | Other paralytic syndrome following nontraumatic subarachnoid hemorrhage affecting right dominant side |
| History of stroke | I69062 | Other paralytic syndrome following nontraumatic subarachnoid hemorrhage affecting left dominant side |
| History of stroke | I69063 | Other paralytic syndrome following nontraumatic subarachnoid hemorrhage affecting right non-dominant side |
| History of stroke | I69064 | Other paralytic syndrome following nontraumatic subarachnoid hemorrhage affecting left non-dominant side |
| History of stroke | I69065 | Other paralytic syndrome following nontraumatic subarachnoid hemorrhage, bilateral |
| History of stroke | I69069 | Other paralytic syndrome following nontraumatic subarachnoid hemorrhage affecting unspecified side |
| History of stroke | I6909 | Other sequelae of nontraumatic subarachnoid hemorrhage |
| History of stroke | I69090 | Apraxia following nontraumatic subarachnoid hemorrhage |
| History of stroke | I69091 | Dysphagia following nontraumatic subarachnoid hemorrhage |
| History of stroke | I69092 | Facial weakness following nontraumatic subarachnoid hemorrhage |
| History of stroke | I69093 | Ataxia following nontraumatic subarachnoid hemorrhage |
| History of stroke | I69098 | Other sequelae following nontraumatic subarachnoid hemorrhage |
| History of stroke | I691 | Sequelae of nontraumatic intracerebral hemorrhage |
| History of stroke | I6910 | Unspecified sequelae of nontraumatic intracerebral hemorrhage |
| History of stroke | I6911 | Cognitive deficits following nontraumatic intracerebral hemorrhage |
| History of stroke | I6912 | Speech and language deficits following nontraumatic intracerebral hemorrhage |
| History of stroke | I69120 | Aphasia following nontraumatic intracerebral hemorrhage |
| History of stroke | I69121 | Dysphasia following nontraumatic intracerebral hemorrhage |
| History of stroke | I69122 | Dysarthria following nontraumatic intracerebral hemorrhage |
| History of stroke | I69123 | Fluency disorder following nontraumatic intracerebral hemorrhage |
| History of stroke | I69128 | Other speech and language deficits following nontraumatic intracerebral hemorrhage |
| History of stroke | I6913 | Monoplegia of upper limb following nontraumatic intracerebral hemorrhage |
| History of stroke | I69131 | Monoplegia of upper limb following nontraumatic intracerebral hemorrhage affecting right dominant side |
| History of stroke | I69132 | Monoplegia of upper limb following nontraumatic intracerebral hemorrhage affecting left dominant side |
| History of stroke | I69133 | Monoplegia of upper limb following nontraumatic intracerebral hemorrhage affecting right non-dominant side |
| History of stroke | I69134 | Monoplegia of upper limb following nontraumatic intracerebral hemorrhage affecting left non-dominant side |
| History of stroke | I69139 | Monoplegia of upper limb following nontraumatic intracerebral hemorrhage affecting unspecified side |
| History of stroke | I6914 | Monoplegia of lower limb following nontraumatic intracerebral hemorrhage |
| History of stroke | I69141 | Monoplegia of lower limb following nontraumatic intracerebral hemorrhage affecting right dominant side |
| History of stroke | I69142 | Monoplegia of lower limb following nontraumatic intracerebral hemorrhage affecting left dominant side |
| History of stroke | I69143 | Monoplegia of lower limb following nontraumatic intracerebral hemorrhage affecting right non-dominant side |
| History of stroke | I69144 | Monoplegia of lower limb following nontraumatic intracerebral hemorrhage affecting left non-dominant side |
| History of stroke | I69149 | Monoplegia of lower limb following nontraumatic intracerebral hemorrhage affecting unspecified side |
| History of stroke | I6915 | Hemiplegia and hemiparesis following nontraumatic intracerebral hemorrhage |
| History of stroke | I69151 | Hemiplegia and hemiparesis following nontraumatic intracerebral hemorrhage affecting right dominant side |
| History of stroke | I69152 | Hemiplegia and hemiparesis following nontraumatic intracerebral hemorrhage affecting left dominant side |

© 2022 Guédon AF et al. *JAMA Network Open.*

| | | |
|---|---|---|
| History of stroke | I69153 | Hemiplegia and hemiparesis following nontraumatic intracerebral hemorrhage affecting right non-dominant side |
| History of stroke | I69154 | Hemiplegia and hemiparesis following nontraumatic intracerebral hemorrhage affecting left non-dominant side |
| History of stroke | I69159 | Hemiplegia and hemiparesis following nontraumatic intracerebral hemorrhage affecting unspecified side |
| History of stroke | I6916 | Other paralytic syndrome following nontraumatic intracerebral hemorrhage |
| History of stroke | I69161 | Other paralytic syndrome following nontraumatic intracerebral hemorrhage affecting right dominant side |
| History of stroke | I69162 | Other paralytic syndrome following nontraumatic intracerebral hemorrhage affecting left dominant side |
| History of stroke | I69163 | Other paralytic syndrome following nontraumatic intracerebral hemorrhage affecting right non-dominant side |
| History of stroke | I69164 | Other paralytic syndrome following nontraumatic intracerebral hemorrhage affecting left non-dominant side |
| History of stroke | I69165 | Other paralytic syndrome following nontraumatic intracerebral hemorrhage, bilateral |
| History of stroke | I69169 | Other paralytic syndrome following nontraumatic intracerebral hemorrhage affecting unspecified side |
| History of stroke | I6919 | Other sequelae of nontraumatic intracerebral hemorrhage |
| History of stroke | I69190 | Apraxia following nontraumatic intracerebral hemorrhage |
| History of stroke | I69191 | Dysphagia following nontraumatic intracerebral hemorrhage |
| History of stroke | I69192 | Facial weakness following nontraumatic intracerebral hemorrhage |
| History of stroke | I69193 | Ataxia following nontraumatic intracerebral hemorrhage |
| History of stroke | I69198 | Other sequelae of nontraumatic intracerebral hemorrhage |
| History of stroke | I692 | Sequelae of other nontraumatic intracranial hemorrhage |
| History of stroke | I6920 | Unspecified sequelae of other nontraumatic intracranial hemorrhage |
| History of stroke | I6921 | Cognitive deficits following other nontraumatic intracranial hemorrhage |
| History of stroke | I6922 | Speech and language deficits following other nontraumatic intracranial hemorrhage |
| History of stroke | I69220 | Aphasia following other nontraumatic intracranial hemorrhage |
| History of stroke | I69221 | Dysphasia following other nontraumatic intracranial hemorrhage |
| History of stroke | I69222 | Dysarthria following other nontraumatic intracranial hemorrhage |
| History of stroke | I69223 | Fluency disorder following other nontraumatic intracranial hemorrhage |
| History of stroke | I69228 | Other speech and language deficits following other nontraumatic intracranial hemorrhage |
| History of stroke | I6923 | Monoplegia of upper limb following other nontraumatic intracranial hemorrhage |
| History of stroke | I69231 | Monoplegia of upper limb following other nontraumatic intracranial hemorrhage affecting right dominant side |
| History of stroke | I69232 | Monoplegia of upper limb following other nontraumatic intracranial hemorrhage affecting left dominant side |
| History of stroke | I69233 | Monoplegia of upper limb following other nontraumatic intracranial hemorrhage affecting right non-dominant side |
| History of stroke | I69234 | Monoplegia of upper limb following other nontraumatic intracranial hemorrhage affecting left non-dominant side |
| History of stroke | I69239 | Monoplegia of upper limb following other nontraumatic intracranial hemorrhage affecting unspecified side |
| History of stroke | I6924 | Monoplegia of lower limb following other nontraumatic intracranial hemorrhage |
| History of stroke | I69241 | Monoplegia of lower limb following other nontraumatic intracranial hemorrhage affecting right dominant side |
| History of stroke | I69242 | Monoplegia of lower limb following other nontraumatic intracranial hemorrhage affecting left dominant side |
| History of stroke | I69243 | Monoplegia of lower limb following other nontraumatic intracranial hemorrhage affecting right non-dominant side |
| History of stroke | I69244 | Monoplegia of lower limb following other nontraumatic intracranial hemorrhage affecting left non-dominant side |
| History of stroke | I69249 | Monoplegia of lower limb following other nontraumatic intracranial hemorrhage affecting unspecified side |
| History of stroke | I6925 | Hemiplegia and hemiparesis following other nontraumatic intracranial hemorrhage |

© 2022 Guédon AF et al. *JAMA Network Open.*

| | | |
|---|---|---|
| History of stroke | I69251 | Hemiplegia and hemiparesis following other nontraumatic intracranial hemorrhage affecting right dominant side |
| History of stroke | I69252 | Hemiplegia and hemiparesis following other nontraumatic intracranial hemorrhage affecting left dominant side |
| History of stroke | I69253 | Hemiplegia and hemiparesis following other nontraumatic intracranial hemorrhage affecting right non-dominant side |
| History of stroke | I69254 | Hemiplegia and hemiparesis following other nontraumatic intracranial hemorrhage affecting left non-dominant side |
| History of stroke | I69259 | Hemiplegia and hemiparesis following other nontraumatic intracranial hemorrhage affecting unspecified side |
| History of stroke | I6926 | Other paralytic syndrome following other nontraumatic intracranial hemorrhage |
| History of stroke | I69261 | Other paralytic syndrome following other nontraumatic intracranial hemorrhage affecting right dominant side |
| History of stroke | I69262 | Other paralytic syndrome following other nontraumatic intracranial hemorrhage affecting left dominant side |
| History of stroke | I69263 | Other paralytic syndrome following other nontraumatic intracranial hemorrhage affecting right non-dominant side |
| History of stroke | I69264 | Other paralytic syndrome following other nontraumatic intracranial hemorrhage affecting left non-dominant side |
| History of stroke | I69265 | Other paralytic syndrome following other nontraumatic intracranial hemorrhage, bilateral |
| History of stroke | I69269 | Other paralytic syndrome following other nontraumatic intracranial hemorrhage affecting unspecified side |
| History of stroke | I6929 | Other sequelae of other nontraumatic intracranial hemorrhage |
| History of stroke | I69290 | Apraxia following other nontraumatic intracranial hemorrhage |
| History of stroke | I69291 | Dysphagia following other nontraumatic intracranial hemorrhage |
| History of stroke | I69292 | Facial weakness following other nontraumatic intracranial hemorrhage |
| History of stroke | I69293 | Ataxia following other nontraumatic intracranial hemorrhage |
| History of stroke | I69298 | Other sequelae of other nontraumatic intracranial hemorrhage |
| History of stroke | I693 | Sequelae of cerebral infarction |
| History of stroke | I6930 | Unspecified sequelae of cerebral infarction |
| History of stroke | I6931 | Cognitive deficits following cerebral infarction |
| History of stroke | I6932 | Speech and language deficits following cerebral infarction |
| History of stroke | I69320 | Aphasia following cerebral infarction |
| History of stroke | I69321 | Dysphasia following cerebral infarction |
| History of stroke | I69322 | Dysarthria following cerebral infarction |
| History of stroke | I69323 | Fluency disorder following cerebral infarction |
| History of stroke | I69328 | Other speech and language deficits following cerebral infarction |
| History of stroke | I6933 | Monoplegia of upper limb following cerebral infarction |
| History of stroke | I69331 | Monoplegia of upper limb following cerebral infarction affecting right dominant side |
| History of stroke | I69332 | Monoplegia of upper limb following cerebral infarction affecting left dominant side |
| History of stroke | I69333 | Monoplegia of upper limb following cerebral infarction affecting right non-dominant side |
| History of stroke | I69334 | Monoplegia of upper limb following cerebral infarction affecting left non-dominant side |
| History of stroke | I69339 | Monoplegia of upper limb following cerebral infarction affecting unspecified side |
| History of stroke | I6934 | Monoplegia of lower limb following cerebral infarction |
| History of stroke | I69341 | Monoplegia of lower limb following cerebral infarction affecting right dominant side |
| History of stroke | I69342 | Monoplegia of lower limb following cerebral infarction affecting left dominant side |
| History of stroke | I69343 | Monoplegia of lower limb following cerebral infarction affecting right non-dominant side |
| History of stroke | I69344 | Monoplegia of lower limb following cerebral infarction affecting left non-dominant side |
| History of stroke | I69349 | Monoplegia of lower limb following cerebral infarction affecting unspecified side |
| History of stroke | I6935 | Hemiplegia and hemiparesis following cerebral infarction |

© 2022 Guédon AF et al. *JAMA Network Open.*

| | | |
|---|---|---|
| History of stroke | I69351 | Hemiplegia and hemiparesis following cerebral infarction affecting right dominant side |
| History of stroke | I69352 | Hemiplegia and hemiparesis following cerebral infarction affecting left dominant side |
| History of stroke | I69353 | Hemiplegia and hemiparesis following cerebral infarction affecting right non-dominant side |
| History of stroke | I69354 | Hemiplegia and hemiparesis following cerebral infarction affecting left non-dominant side |
| History of stroke | I69359 | Hemiplegia and hemiparesis following cerebral infarction affecting unspecified side |
| History of stroke | I6936 | Other paralytic syndrome following cerebral infarction |
| History of stroke | I69361 | Other paralytic syndrome following cerebral infarction affecting right dominant side |
| History of stroke | I69362 | Other paralytic syndrome following cerebral infarction affecting left dominant side |
| History of stroke | I69363 | Other paralytic syndrome following cerebral infarction affecting right non-dominant side |
| History of stroke | I69364 | Other paralytic syndrome following cerebral infarction affecting left non-dominant side |
| History of stroke | I69365 | Other paralytic syndrome following cerebral infarction, bilateral |
| History of stroke | I69369 | Other paralytic syndrome following cerebral infarction affecting unspecified side |
| History of stroke | I6939 | Other sequelae of cerebral infarction |
| History of stroke | I69390 | Apraxia following cerebral infarction |
| History of stroke | I69391 | Dysphagia following cerebral infarction |
| History of stroke | I69392 | Facial weakness following cerebral infarction |
| History of stroke | I69393 | Ataxia following cerebral infarction |
| History of stroke | I69398 | Other sequelae of cerebral infarction |
| History of stroke | I698 | Sequelae of other cerebrovascular diseases |
| History of stroke | I6980 | Unspecified sequelae of other cerebrovascular disease |
| History of stroke | I6981 | Cognitive deficits following other cerebrovascular disease |
| History of stroke | I6982 | Speech and language deficits following other cerebrovascular disease |
| History of stroke | I69820 | Aphasia following other cerebrovascular disease |
| History of stroke | I69821 | Dysphasia following other cerebrovascular disease |
| History of stroke | I69822 | Dysarthria following other cerebrovascular disease |
| History of stroke | I69823 | Fluency disorder following other cerebrovascular disease |
| History of stroke | I69828 | Other speech and language deficits following other cerebrovascular disease |
| History of stroke | I6983 | Monoplegia of upper limb following other cerebrovascular disease |
| History of stroke | I69831 | Monoplegia of upper limb following other cerebrovascular disease affecting right dominant side |
| History of stroke | I69832 | Monoplegia of upper limb following other cerebrovascular disease affecting left dominant side |
| History of stroke | I69833 | Monoplegia of upper limb following other cerebrovascular disease affecting right non-dominant side |
| History of stroke | I69834 | Monoplegia of upper limb following other cerebrovascular disease affecting left non-dominant side |
| History of stroke | I69839 | Monoplegia of upper limb following other cerebrovascular disease affecting unspecified side |
| History of stroke | I6984 | Monoplegia of lower limb following other cerebrovascular disease |
| History of stroke | I69841 | Monoplegia of lower limb following other cerebrovascular disease affecting right dominant side |
| History of stroke | I69842 | Monoplegia of lower limb following other cerebrovascular disease affecting left dominant side |
| History of stroke | I69843 | Monoplegia of lower limb following other cerebrovascular disease affecting right non-dominant side |
| History of stroke | I69844 | Monoplegia of lower limb following other cerebrovascular disease affecting left non-dominant side |
| History of stroke | I69849 | Monoplegia of lower limb following other cerebrovascular disease affecting unspecified side |
| History of stroke | I6985 | Hemiplegia and hemiparesis following other cerebrovascular disease |

© 2022 Guédon AF et al. *JAMA Network Open.*

| | | |
|---|---|---|
| History of stroke | I69851 | Hemiplegia and hemiparesis following other cerebrovascular disease affecting right dominant side |
| History of stroke | I69852 | Hemiplegia and hemiparesis following other cerebrovascular disease affecting left dominant side |
| History of stroke | I69853 | Hemiplegia and hemiparesis following other cerebrovascular disease affecting right non-dominant side |
| History of stroke | I69854 | Hemiplegia and hemiparesis following other cerebrovascular disease affecting left non-dominant side |
| History of stroke | I69859 | Hemiplegia and hemiparesis following other cerebrovascular disease affecting unspecified side |
| History of stroke | I6986 | Other paralytic syndrome following other cerebrovascular disease |
| History of stroke | I69861 | Other paralytic syndrome following other cerebrovascular disease affecting right dominant side |
| History of stroke | I69862 | Other paralytic syndrome following other cerebrovascular disease affecting left dominant side |
| History of stroke | I69863 | Other paralytic syndrome following other cerebrovascular disease affecting right non-dominant side |
| History of stroke | I69864 | Other paralytic syndrome following other cerebrovascular disease affecting left non-dominant side |
| History of stroke | I69865 | Other paralytic syndrome following other cerebrovascular disease, bilateral |
| History of stroke | I69869 | Other paralytic syndrome following other cerebrovascular disease affecting unspecified side |
| History of stroke | I6989 | Other sequelae of other cerebrovascular disease |
| History of stroke | I69890 | Apraxia following other cerebrovascular disease |
| History of stroke | I69891 | Dysphagia following other cerebrovascular disease |
| History of stroke | I69892 | Facial weakness following other cerebrovascular disease |
| History of stroke | I69893 | Ataxia following other cerebrovascular disease |
| History of stroke | I69898 | Other sequelae of other cerebrovascular disease |
| History of stroke | I699 | Sequelae of unspecified cerebrovascular diseases |
| History of stroke | I6990 | Unspecified sequelae of unspecified cerebrovascular disease |
| History of stroke | I6991 | Cognitive deficits following unspecified cerebrovascular disease |
| History of stroke | I6992 | Speech and language deficits following unspecified cerebrovascular disease |
| History of stroke | I69920 | Aphasia following unspecified cerebrovascular disease |
| History of stroke | I69921 | Dysphasia following unspecified cerebrovascular disease |
| History of stroke | I69922 | Dysarthria following unspecified cerebrovascular disease |
| History of stroke | I69923 | Fluency disorder following unspecified cerebrovascular disease |
| History of stroke | I69928 | Other speech and language deficits following unspecified cerebrovascular disease |
| History of stroke | I6993 | Monoplegia of upper limb following unspecified cerebrovascular disease |
| History of stroke | I69931 | Monoplegia of upper limb following unspecified cerebrovascular disease affecting right dominant side |
| History of stroke | I69932 | Monoplegia of upper limb following unspecified cerebrovascular disease affecting left dominant side |
| History of stroke | I69933 | Monoplegia of upper limb following unspecified cerebrovascular disease affecting right non-dominant side |
| History of stroke | I69934 | Monoplegia of upper limb following unspecified cerebrovascular disease affecting left non-dominant side |
| History of stroke | I69939 | Monoplegia of upper limb following unspecified cerebrovascular disease affecting unspecified side |
| History of stroke | I6994 | Monoplegia of lower limb following unspecified cerebrovascular disease |
| History of stroke | I69941 | Monoplegia of lower limb following unspecified cerebrovascular disease affecting right dominant side |
| History of stroke | I69942 | Monoplegia of lower limb following unspecified cerebrovascular disease affecting left dominant side |
| History of stroke | I69943 | Monoplegia of lower limb following unspecified cerebrovascular disease affecting right non-dominant side |
| History of stroke | I69944 | Monoplegia of lower limb following unspecified cerebrovascular disease affecting left non-dominant side |
| History of stroke | I69949 | Monoplegia of lower limb following unspecified cerebrovascular disease affecting unspecified side |
| History of stroke | I6995 | Hemiplegia and hemiparesis following unspecified cerebrovascular disease |

© 2022 Guédon AF et al. *JAMA Network Open.*

| | | |
|---|---|---|
| History of stroke | I69951 | Hemiplegia and hemiparesis following unspecified cerebrovascular disease affecting right dominant side |
| History of stroke | I69952 | Hemiplegia and hemiparesis following unspecified cerebrovascular disease affecting left dominant side |
| History of stroke | I69953 | Hemiplegia and hemiparesis following unspecified cerebrovascular disease affecting right non-dominant side |
| History of stroke | I69954 | Hemiplegia and hemiparesis following unspecified cerebrovascular disease affecting left non-dominant side |
| History of stroke | I69959 | Hemiplegia and hemiparesis following unspecified cerebrovascular disease affecting unspecified side |
| History of stroke | I6996 | Other paralytic syndrome following unspecified cerebrovascular disease |
| History of stroke | I69961 | Other paralytic syndrome following unspecified cerebrovascular disease affecting right dominant side |
| History of stroke | I69962 | Other paralytic syndrome following unspecified cerebrovascular disease affecting left dominant side |
| History of stroke | I69963 | Other paralytic syndrome following unspecified cerebrovascular disease affecting right non-dominant side |
| History of stroke | I69964 | Other paralytic syndrome following unspecified cerebrovascular disease affecting left non-dominant side |
| History of stroke | I69965 | Other paralytic syndrome following unspecified cerebrovascular disease, bilateral |
| History of stroke | I69969 | Other paralytic syndrome following unspecified cerebrovascular disease affecting unspecified side |
| History of stroke | I6999 | Other sequelae of unspecified cerebrovascular disease |
| History of stroke | I69990 | Apraxia following unspecified cerebrovascular disease |
| History of stroke | I69991 | Dysphagia following unspecified cerebrovascular disease |
| History of stroke | I69992 | Facial weakness following unspecified cerebrovascular disease |
| History of stroke | I69993 | Ataxia following unspecified cerebrovascular disease |
| History of stroke | I69998 | Other sequelae following unspecified cerebrovascular disease |
| Arterial hypertension | I10 | Essential (primary) hypertension |
| Arterial hypertension | I11 | Hypertensive heart disease |
| Arterial hypertension | I110 | Hypertensive heart disease with heart failure |
| Arterial hypertension | I119 | Hypertensive heart disease without heart failure |
| Arterial hypertension | I12 | Hypertensive chronic kidney disease |
| Arterial hypertension | I120 | Hypertensive chronic kidney disease with stage 5 chronic kidney disease or end stage renal disease |
| Arterial hypertension | I129 | Hypertensive chronic kidney disease with stage 1 through stage 4 chronic kidney disease, or unspecified chronic kidney disease |
| Arterial hypertension | I13 | Hypertensive heart and chronic kidney disease |
| Arterial hypertension | I130 | Hypertensive heart and chronic kidney disease with heart failure and stage 1 through stage 4 chronic kidney disease, or unspecified chronic kidney disease |
| Arterial hypertension | I131 | Hypertensive heart and chronic kidney disease without heart failure |
| Arterial hypertension | I1310 | Hypertensive heart and chronic kidney disease without heart failure, with stage 1 through stage 4 chronic kidney disease, or unspecified chronic kidney disease |
| Arterial hypertension | I1311 | Hypertensive heart and chronic kidney disease without heart failure, with stage 5 chronic kidney disease, or end stage renal disease |
| Arterial hypertension | I132 | Hypertensive heart and chronic kidney disease with heart failure and with stage 5 chronic kidney disease, or end stage renal disease |
| Arterial hypertension | I15 | Secondary hypertension |
| Arterial hypertension | I150 | Renovascular hypertension |
| Arterial hypertension | I151 | Hypertension secondary to other renal disorders |
| Arterial hypertension | I152 | Hypertension secondary to endocrine disorders |

© 2022 Guédon AF et al. *JAMA Network Open.*

| | | |
|---|---|---|
| Arterial hypertension | I158 | Other secondary hypertension |
| Arterial hypertension | I159 | Secondary hypertension, unspecified |
| Former smoker | F1720 | Tobacco dependence syndrome, currently abstinent |
| Former smoker | F1721 | Tobacco dependence syndrome, currently abstinent but in a protected environment |
| Former smoker | F1722 | Tobacco dependence syndrome, currently on a maintenance or substitution diet under medical supervision |
| Former smoker | F1723 | Tobacco dependence syndrome, currently abstinent, but taking aversive or blocking medications |
| Former smoker | Z864 | Personal history of substance abuse (former smoker) |
| Current smoker | F170 | Mental and behavioral disorders related to tobacco use: acute intoxication |
| Current smoker | F1700 | Mental and behavioural disorders due to acute tobacco intoxication without complications |
| Current smoker | F1701 | Mental and behavioural disorders due to acute tobacco intoxication with trauma or other physical injury |
| Current smoker | F1702 | Mental and behavioral disorders due to acute tobacco intoxication with other medical complications |
| Current smoker | F1703 | Mental and behavioral disorders due to acute tobacco intoxication, with delirium |
| Current smoker | F1704 | Mental and behavioral disorders due to acute tobacco intoxication, with perceptual distortion |
| Current smoker | F1705 | Mental and behavioural disorders due to acute tobacco intoxication, with coma |
| Current smoker | F1706 | Mental and behavioural disorders due to acute tobacco intoxication, with convulsions |
| Current smoker | F1707 | Mental and behavioural disorders due to acute pathological tobacco intoxication |
| Current smoker | F171 | Mental and behavioural disorders related to tobacco use: harmful use |
| Current smoker | F1724 | Tobacco dependence syndrome, current use |
| Current smoker | F17240 | Tobacco dependence syndrome, current use, no physical symptoms |
| Current smoker | F17241 | Tobacco dependence syndrome, current use, with physical symptoms |
| Current smoker | F1725 | Tobacco dependence syndrome, ongoing use |
| Current smoker | F1726 | Tobacco dependence syndrome, episodic use |
| Current smoker | F173 | Mental and behavioral disorders related to tobacco use: withdrawal syndrome |
| Current smoker | F1730 | Smoking withdrawal syndrome, uncomplicated |
| Current smoker | F1731 | Smoking withdrawal syndrome, with seizures |
| Current smoker | F174 | Mental and behavioural disorders related to tobacco use: withdrawal syndrome with delirium |
| Current smoker | F1740 | Smoking withdrawal syndrome with delirium, without convulsion |
| Current smoker | F1741 | Smoking withdrawal syndrome with delirium, with convulsions |
| Current smoker | F175 | Mental and behavioural disorders related to tobacco use: psychotic disorder |
| Current smoker | F1750 | Psychotic disorder with schizophrenic features, due to tobacco use |
| Current smoker | F1751 | Psychotic disorder with delusions in the foreground, due to tobacco |
| Current smoker | F1752 | Psychotic disorder with prominent hallucinations due to tobacco |
| Current smoker | F1753 | Psychotic disorder with prominent polymorphic symptoms, due to smoking |
| Current smoker | F1754 | Psychotic disorder with prominent depressive symptoms due to smoking |
| Current smoker | F1755 | Psychotic disorder with manic symptoms in the foreground, due to smoking |
| Current smoker | F1756 | Mixed psychotic disorder, due to smoking |
| Current smoker | F176 | Mental and behavioural disorders related to tobacco use: amnestic syndrome |
| Current smoker | F177 | Mental and behavioural disorders related to tobacco use: late-onset residual or psychotic disorder |
| Current smoker | F1770 | Flashbacks, due to smoking |
| Current smoker | F1771 | Residual or late-onset personality or behavioral disorder due to tobacco |
| Current smoker | F1772 | Residual or late-onset psychotic disorder due to tobacco |
| Current smoker | F1773 | Dementia, due to smoking |
| Current smoker | F1774 | Other persistent or late-onset cognitive impairment due to tobacco |
| Current smoker | F1775 | Late-onset psychotic disorder, due to tobacco |
| Current smoker | F178 | Other mental and behavioral disorders related to tobacco use |

© 2022 Guédon AF et al. *JAMA Network Open.*

| | | |
|---|---|---|
| Current smoker | F179 | Unspecified mental and behavioral disorders related to tobacco use |
| Current smoker | T652 | Toxic effect of tobacco and nicotine |
| Current smoker | Z587 | Exposure to tobacco smoke |
| Current smoker | Z720 | Difficulties related to tobacco use (without abuse) |
| Dyslipidemia | E78 | Disorders of lipoprotein metabolism and other lipidemias |
| Dyslipidemia | E780 | Pure hypercholesterolemia |
| Dyslipidemia | E781 | Pure hyperglyceridemia |
| Dyslipidemia | E782 | Mixed hyperlipidemia |
| Dyslipidemia | E783 | Hyperchylomicronemia |
| Dyslipidemia | E784 | Other hyperlipidemia |
| Dyslipidemia | E785 | Hyperlipidemia, unspecified |
| Dyslipidemia | E786 | Lipoprotein deficiency |
| Dyslipidemia | E788 | Other disorders of lipoprotein metabolism |
| Dyslipidemia | E789 | Disorder of lipoprotein metabolism, unspecified |
| Diabetes mellitus | E10 | Type 1 diabetes mellitus |
| Diabetes mellitus | E101 | Type 1 diabetes mellitus with ketoacidosis |
| Diabetes mellitus | E102 | Type 1 diabetes mellitus with kidney complications |
| Diabetes mellitus | E103 | Type 1 diabetes mellitus with ophthalmic complications |
| Diabetes mellitus | E104 | Type 1 diabetes mellitus with neurological complications |
| Diabetes mellitus | E105 | Type 1 diabetes mellitus with circulatory complications |
| Diabetes mellitus | E106 | Type 1 diabetes mellitus with other specified complications |
| Diabetes mellitus | E108 | Type 1 diabetes mellitus with unspecified complications |
| Diabetes mellitus | E109 | Type 1 diabetes mellitus without complications |
| Diabetes mellitus | E11 | Type 2 diabetes mellitus |
| Diabetes mellitus | E110 | Type 2 diabetes mellitus with hyperosmolarity |
| Diabetes mellitus | E1100 | Type 2 diabetes mellitus with hyperosmolarity without nonketotic hyperglycemic-hyperosmolar coma (NKHHC) |
| Diabetes mellitus | E112 | Type 2 diabetes mellitus with kidney complications |
| Diabetes mellitus | E113 | Type 2 diabetes mellitus with ophthalmic complications |
| Diabetes mellitus | E114 | Type 2 diabetes mellitus with neurological complications |
| Diabetes mellitus | E1140 | Type 2 diabetes mellitus with diabetic neuropathy, unspecified |
| Diabetes mellitus | E115 | Type 2 diabetes mellitus with circulatory complications |
| Diabetes mellitus | E116 | Type 2 diabetes mellitus with other specified complications |
| Diabetes mellitus | E118 | Type 2 diabetes mellitus with unspecified complications |
| Diabetes mellitus | E119 | Type 2 diabetes mellitus without complications |
| Diabetes mellitus | E13 | Other specified diabetes mellitus |
| Diabetes mellitus | E130 | Other specified diabetes mellitus with hyperosmolarity |
| Diabetes mellitus | E131 | Other specified diabetes mellitus with ketoacidosis |
| Diabetes mellitus | E132 | Other specified diabetes mellitus with kidney complications |
| Diabetes mellitus | E133 | Other specified diabetes mellitus with ophthalmic complications |
| Diabetes mellitus | E134 | Other specified diabetes mellitus with neurological complications |
| Diabetes mellitus | E135 | Other specified diabetes mellitus with circulatory complications |
| Diabetes mellitus | E136 | Other specified diabetes mellitus with other specified complications |
| Diabetes mellitus | E138 | Other specified diabetes mellitus with unspecified complications |
| Diabetes mellitus | E139 | Other specified diabetes mellitus without complications |

© 2022 Guédon AF et al. *JAMA Network Open.*

**eMethods 2.** List of CCAM (French Common Classification of Medical Procedures) Used for Outcomes Definitions

| Outcome | CCAM code | Label |
|---|---|---|
| Major amputation | NZFA002 | Transtibial amputation |
| | NZFA005 | Amputation or disarticulation of the midfoot or forefoot, without stabilization of the hindfoot |
| | NZFA007 | Transfemoral amputation |
| | NZFA013 | Amputation or disarticulation of the midfoot or forefoot, with stabilization of the hindfoot |
| Peripheral endovascular revascularization | DGPF001 | Percutaneous arterial desobstruction of the aortic bifurcation |
| | DGPF002 | Recanalization of the aortic bifurcation with stenting by bilateral percutaneous arterial approach |
| | EDPF006 | Recanalization of the common iliac artery and/or external iliac artery with covered stenting, by percutaneous arterial approach |
| | EDPF008 | Recanalization of the common iliac artery and/or external iliac artery without stenting, by percutaneous arterial approach |
| | EDPF009 | Recanalization of the common iliac artery and/or the external iliac artery with stenting, by percutaneous arterial approach |
| | EEAF001 | Intraluminal dilatation of several arteries of the lower limb without stenting, by percutaneous arterial approach |
| | EEAF002 | Intraluminal dilatation of a lower limb artery with intraluminal dilatation of the common iliac artery and/or the homolateral external iliac artery with stenting, by percutaneous arterial approach |
| | EEAF003 | Intraluminal dilatation of a lower limb artery without stenting, by percutaneous arterial approach |
| | EEAF004 | Intraluminal dilatation of a lower limb artery with percutaneous arterial stenting |
| | EEAF005 | Intraluminal dilatation of a lower limb artery with intraluminal dilatation of the common iliac artery and/or the homolateral external iliac artery without stenting, by percutaneous arterial approach |
| | EEAF006 | Intraluminal dilatation of several arteries of the lower limb with stenting, by percutaneous arterial approach |
| | EEJF001 | Percutaneous arterial thromboaspiration of the artery or bypass of the lower limb |
| | EENF001 | Superselective in situ fibrinolysis of an artery or arterial bypass of the lower limb, by percutaneous arterial approach |
| | EENF002 | Selective or superselective in situ fibrinolysis of an artery or an arterial bypass of the lower limb, by percutaneous arterial approach |
| | EEPF001 | Recanalization of a lower limb artery with percutaneous arterial stenting |
| | EEPF002 | Recanalization of a lower limb artery without stenting, by percutaneous arterial approach |
| | ENAF001 | Intraluminal dilatation of a non-anatomic limb artery bypass graft with percutaneous arterial stenting |
| | ENAF002 | Intraluminal dilatation of a non-anatomic limb artery bypass graft without stenting by percutaneous arterial approach |
| Peripheral surgical revascularization | DGFA001 | Thromboendarterectomy of the trunk of the abdominal aorta, by laparotomy |
| | DGFA003 | Aortobiiliac thromboendarterectomy by laparotomy |
| | DGFA004 | Thrombectomy of the abdominal aorta, common iliac artery and/or external iliac artery through bilateral inguinofemoral approach |
| | DGFA005 | Thrombectomy of a bypass or thromboendarterectomy of the abdominal aorta or its branches by laparotomy |
| | DGFA007 | Unilateral aorto-ilio-femoral thromboendarterectomy by laparotomy |
| | DGFA008 | Bilateral aorto-ilio-femoral thromboendarterectomy, by laparotomy |
| | DGFA009 | Unilateral aorto-iliac thromboendarterectomy, by laparotomy |
| | DGFA010 | Thrombectomy of the abdominal aorta, common iliac artery and/or external iliac artery by laparotomy |

© 2022 Guédon AF et al. *JAMA Network Open.*

| | | |
|---|---|---|
| | DGFA012 | Thrombectomy of a bypass or thromboendarterectomy of the abdominal aorta or its branches through a inguinofemoral approach |
| | EDFA003 | Ilio-femoral thromboendarterectomy, by laparotomy |
| | EDFA006 | Iliac thromboendarterectomy, by laparotomy |
| | EDFA007 | Iliofemoral thromboendarterectomy, by direct inguinofemoral approach |
| | DGCA004 | Bifurcated aortobifemoral bypass, by laparotomy with infrarenal clamping |
| | DGCA007 | Infrarenal aortaortic bypass by laparotomy, with infrarenal clamping |
| | DGCA009 | Unilateral aortofemoral bypass, by laparotomy with infrarenal clamping |
| | DGCA010 | Bifurcated aortobifemoral bypass surgery with suprarenal clamping |
| | DGCA012 | Infrarenal aortaortic bypass by laparotomy, with suprarenal clamping |
| | DGCA019 | Unilateral aortofemoral bypass, by laparotomy with suprarenal clamping |
| | DGCA020 | Aorto-ilio-femoral bifurcated bypass surgery with suprarenal clamping |
| | DGCA021 | Aortobirenal bypass surgery, by laparotomy |
| | DGCA022 | Bifurcated aortobisilic bypass, by laparotomy with infrarenal clamping |
| | DGCA026 | Bifurcated aortobisilic bypass, by laparotomy with suprarenal clamping |
| | DGCA029 | Aorto-ilio-femoral bifurcated bypass surgery with infrarenal clamping |
| | DGCA030 | Iterative bifurcated aortobifemoral bypass [redux] without prosthesis removal, by laparotomy |
| | EDCA003 | Ilio-iliac, iliofemoral or femorofemoral artery bypass grafting using a direct approach |
| | EEFA001 | Thromboendarterectomy of the femoral artery and/or its branches, by direct approach |
| | EEFA002 | Thrombectomy of the lower limb artery, by popliteal approach |
| | EEFA003 | Thromboendarterectomy of the popliteal artery, by direct approach |
| | EEFA004 | Thrombectomy of the lower limb artery, by inguinofemoral approach |
| | EDCA004 | Ilio-femoral arterial bypass for anastomotic complication on femoral bifurcation prosthesis, by direct approach |
| | EDCA005 | Homolateral iliofemoral artery bypass surgery, direct approach |
| | EECA001 | Femoropopliteal artery bypass graft above the knee joint space using a direct approach |
| | EECA002 | Homolateral femorofemoral artery bypass surgery, direct approach |
| | EECA003 | Femoropopliteal artery bypass below the knee joint space, using a direct approach |
| | EECA005 | Bypass surgery of a foot artery, by direct approach |
| | EECA006 | Subclaviofemoral or axillobifemoral arterial bypass through direct approach |
| | EECA007 | Subclaviofemoral or axillofemoral arterial bypass through a direct approach |
| | EECA008 | Femorotibial or femoroperoneal arterial bypass surgery without a venous collar, using a direct approach |
| | EECA010 | Femorotibial or femoroperoneal arterial bypass with venous collar, by direct approach |
| | EECA012 | Multiple staged [sequential] or bifurcated bypass surgery of the lower limb arteries, using a direct approach |
| | EEAA002 | Enlargement angioplasty of a lower limb arterial bypass by direct approach |

© 2022 Guédon AF et al. *JAMA Network Open.*
