# Supplementary material for: Association of Lipoprotein(a) Levels With Incidence of Major Adverse Limb Events
Source: JAMA Netw Open. 2022 Dec 8;5(12):e2245720. doi: 10.1001/jamanetworkopen.2022.45720 (PMC9856359; doi:10.1001/jamanetworkopen.2022.45720)
Supplement: Supplement 2. — Data Sharing Statement [file jamanetwopen-e2245720-s002.pdf]

Data Sharing Statement

Guédon. Association of Lipoprotein(a) Levels With Incidence of Major Adverse Limb Events. *JAMA Netw Open.* Published December 08, 2022. doi:10.1001/jamanetworkopen.2022.45720

Data
Data available: Yes
Data types: Deidentified participant data, Data dictionary
How to access data: Data will be made available upon request to the correponding author: guillaume.goudot@gmail.com
When available: With publication

Supporting Documents
Document types: None

Additional Information
Who can access the data: Anyone requesting the data
Types of analyses: For a research purpose
Mechanisms of data availability: Data will be made available after a signed data access agreement
